# Universal Method for Covalent Attachment of Hydrogels to Diverse Polymeric Surfaces for Biomedical Applications

**DOI:** 10.1002/adma.202503524

**Published:** 2025-08-07

**Authors:** Masoud Zhianmanesh, Azin Khodaei, Matthew Crago, Oliver Lotz, Sina Naficy, Fariba Dehghani, Marcela Bilek, Saber Amin Yavari, Behnam Akhavan

**Affiliations:** ^1^ School of Biomedical Engineering The University of Sydney Sydney NSW 2006 Australia; ^2^ School of Engineering The University of Newcastle Callaghan NSW 2308 Australia; ^3^ Department of Orthopedics Utrecht 3584 The Netherlands; ^4^ School of Chemical and Biomolecular Engineering The University of Sydney Sydney NSW 2006 Australia; ^5^ The University of Sydney Nano Institute The University of Sydney Sydney NSW 2006 Australia; ^6^ School of Physics The University of Sydney Sydney NSW 2006 Australia; ^7^ Charles Perkins Centre The University of Sydney Sydney NSW 2006 Australia; ^8^ Regenerative Medicine Center Utrecht University Medical Centre Utrecht Utrecht 3584 The Netherlands; ^9^ Hunter Medical Research Institute (HMRI) Precision Medicine Research Program New Lambton Heights NSW 2305 Australia

**Keywords:** hydrogel coatings, hydrogel immobilization, plasma surface functionalization, solid‐hydrogel constructs

## Abstract

Hydrogels, renowned for their biocompatibility and capacity to mimic biological tissues, are integral to many biomedical applications, such as implantable devices and wound dressings. However, their poor mechanical strength and the challenge of achieving durable adhesion to polymeric surfaces have hindered their broader utility. Current methods of creating hybrid solid‐hydrogel (HSH) structures often rely on complex chemical linkers, adding steps, cytotoxic risks, and scalability issues. Here, a novel, reagent‐free method that covalently bonds hydrogels to polymeric substrates directly via reactive oxygen species (ROS) generated by an atmospheric pressure plasma jet (APPJ) is introduced. Through an evaporation‐induced enhanced concentration (EIEC) approach, robust hydrogel layers are formed on ROS‐functionalized surfaces, eliminating the need for silane‐based linkers and achieving up to 60 kPa adhesion strength in wet conditions. This strategy offers robust hydrogel adhesion, reduces processing complexity, and preserves cytocompatibility, as demonstrated by the culture of human mesenchymal stem cells (hMSCs) and THP‐1 derived macrophages with minimal immune response. Applicable across various hydrogels, such as gelatin methacryloyl (GelMA), chitosan, and polymeric substrates, including Teflon, polyethylene, and polycaprolactone (PCL), this dry process holds substantial promise for integration into advanced biomanufacturing systems, such as 3D bioprinters, unlocking new potentials in tissue engineering and biomedical device fabrication.

## Graphical abstract

A universal, reagent‐free strategy is presented for covalently attaching hydrogels to diverse polymeric substrates through reactive oxygen species. The scalable, linker‐free approach enables robust adhesion and broad material compatibility, advancing the fabrication of hybrid solid–hydrogel systems for next‐generation biomedical devices and bioprinting applications.

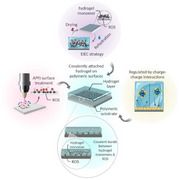

## 1 Introduction

Hydrogels, a versatile class of soft materials, are widely employed in tissue engineering due to their remarkable mechanobiological properties, including hydrophilicity, biodegradability, and superabsorbability.^[^ ^1^, ^2^ ^]^ Hydrogels can mimic intricate extracellular matrixes analogous to native tissues, thereby promoting favorable cellular responses. However, standalone hydrogel‐based constructs suffer from poor mechanical strength and a lack of structural integrity, which significantly restricts their efficacy in a wide range of applications, including wound dressings,^[^ ^3^, ^4^ ^]^ implantable devices,^[^ ^5^ ^]^ diagnostic tools,^[^ ^6^ ^]^ soft robotics,^[^ ^7^ ^]^ and 3D bioprinting.^[^ ^8^ ^]^ A viable solution is to use solid materials as supporting substrates, providing enhanced structural integrity.^[^ ^9^, ^10^, ^11^, ^12^ ^]^ The resulting hybrid solid‐hydrogel (HSH) structures, thus, benefit from both the robustness of the solid substrate and the cell‐supportive properties of hydrogels. Such hybrid biomaterials hold significant potential for diverse biomedical applications such as tissue regeneration, diagnostic kits, drug delivery, and 3D cell culture platforms.^[^ ^10^, ^13^, ^14^, ^15^, ^16^, ^17^ ^]^ For almost all of these applications, hydrogels must be robustly anchored to solid surfaces to maintain their functionality and prevent coating delamination under harsh physiological conditions.^[^ ^18^ ^]^ Nevertheless, a long‐lasting challenge is the limited capacity of hydrogels to effectively interact with and adhere to most solid materials, rendering them impractical as durable coatings.^[^ ^10^, ^19^, ^20^ ^]^ For hydrogels to be stable on solid surfaces, robust interfacial adhesion is imperative, which can be achieved by forming covalent bonds between hydrogel chains and surfaces.^[^ ^10^, ^11^, ^19^ ^]^ Recent advancements in this domain are focused on modification techniques, including physical and chemical alterations to either hydrogel matrices, solid surfaces, or both, aiming to create durable interfaces.^[^ ^21^ ^]^

Modification of hydrogels involves the conjugation of various functional groups, including N‐Hydroxysuccinimide (NHS), hydroxyl, and catechol groups, to hydrogel chains.^[^ ^11^, ^22^, ^23^, ^24^ ^]^ These functional groups enhance the adhesiveness or toughness of the hydrogels, facilitating the formation of covalent bonds with the underlying solid surfaces. However, such chemically modified hydrogels may exhibit undesired adhesion to adjacent tissues or other surfaces^[^ ^25^ ^]^ and often require high monomer concentrations, often exceeding 20% (w/v) for tough hydrogels, which can negatively affect cellular behavior.^[^ ^26^, ^27^, ^28^ ^]^

Surface modification of solid substrates typically relies on coupling reagents like N‐(3‐Dimethylaminopropyl)‐N'‐ethylcarbodiimide and NHS^[^ ^10^ ^]^ and/or chemical linkers, including silane compounds.^[^ ^11^, ^29^ ^]^ For instance, Yuk et al.^[^ ^11^ ^]^ used silane compounds to covalently bond chemically modified hydrogels on diverse nonpolymeric solid surfaces. However, these methods rely on multi‐step wet chemical processes to fabricate tough hydrogels and involve the introduction of an intermediary layer of molecules such as silane 3‐(trimethoxysilyl)propyl methacrylate (TMSPMA) and (3‐aminopropyl) triethoxysilane (APTES).^[^ ^11^, ^20^ ^]^ As such, a technology that can achieve a robust hydrogel‐solid conjugate structure while minimizing complexity, cytotoxicity, and scalability issues, bypassing the need for chemical linkers and hydrogel modifications altogether, is highly sought after.

Direct formation of covalent bonds between the hydrogel molecules and solid surfaces offers a promising solution. We have recently demonstrated that radical‐functionalized surfaces, created using a plasma immersion ion implantation (PIII) technique, enable direct, reagent‐free, covalent attachment of hydrogels to solid polymeric substrates.^[^ ^12^ ^]^ However, while eliminating the need for wet chemical processes, this approach relies on vacuum systems, which can be restrictive in certain settings. For example, its integration with 3D bioprinters in the rapidly evolving area of additive bio‐manufacturing is not feasible.^[^ ^30^ ^]^ Our team has developed a reagent‐free method for direct covalent attachment of proteins and polyacrylamide hydrogels to polymeric surfaces functionalized by atmospheric pressure plasma.^[^ ^31^, ^32^, ^33^ ^]^ Most recently, we have shown that nonradical reactive oxygen species (ROS) generated on the surfaces of polymeric substrates allow for direct covalent attachment of proteins on activated surfaces.^[^ ^34^ ^]^ Building on these findings, we hypothesize that biomolecule‐based hydrogels can covalently bond to ROS‐functionalized surfaces.

**Figure 1:**
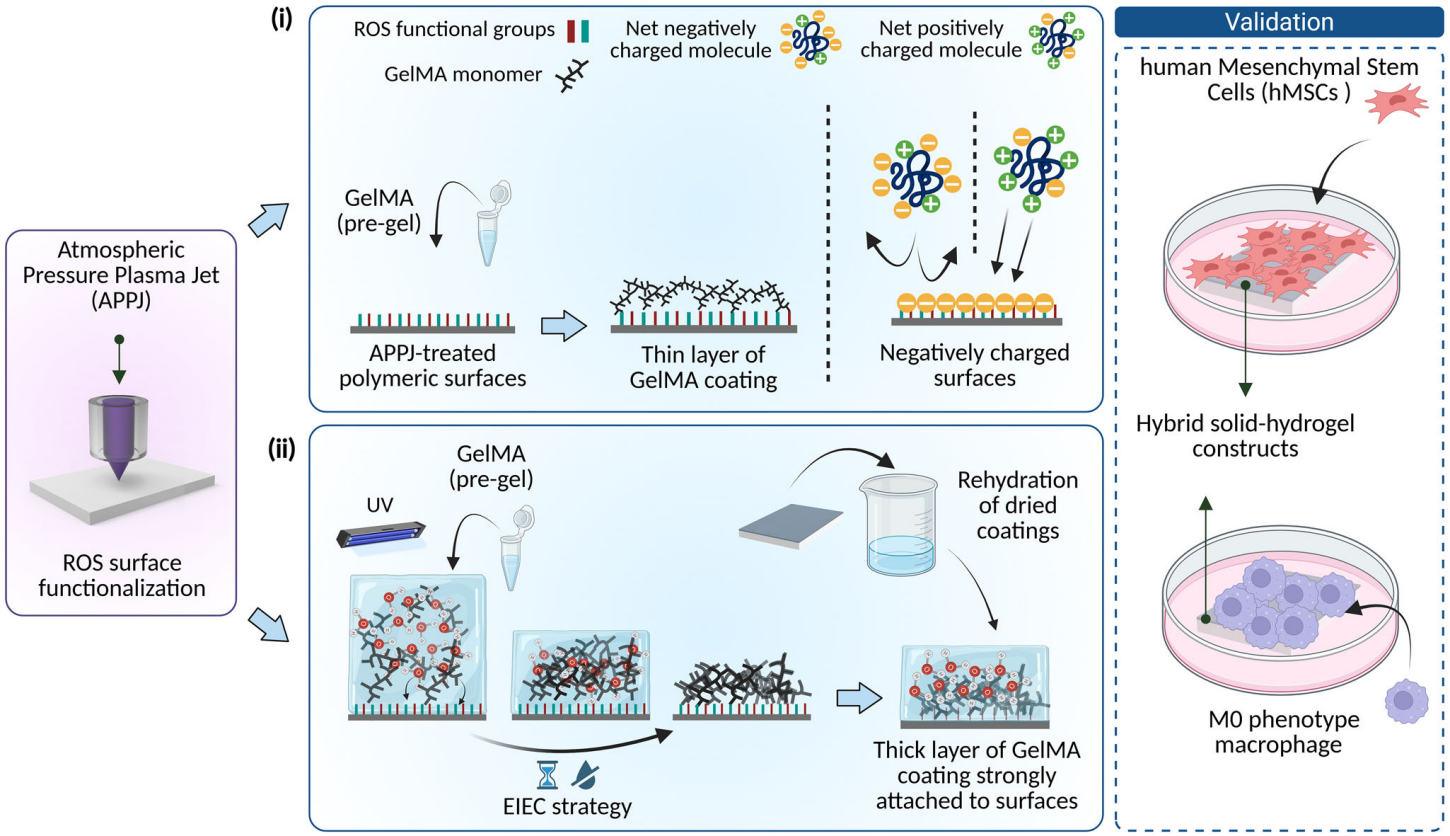
Fabrication of hybrid solid‐hydrogel (HSH) constructs using atmospheric pressure plasma jet (APPJ) combined with an evaporation‐induced enhanced concentration (EIEC) strategy. (i) Gelatin methacryloyl (GelMA) monomers are covalently attached to ROS‐functionalized (reactive oxygen species‐functionalized) polymeric surfaces, forming a monolayer. APPJ treatment adds oxygen‐containing functional groups (OFGs), including ROS, on polymeric surfaces, making them negatively charged and capable of attracting molecules with positive charges. (ii) Thicker GelMA coatings were achieved using the EIEC strategy. Cell studies confirmed the biocompatibility of the HSH constructs and assessed immune responses by culturing human mesenchymal stem cells (hMSCs) and M0 phenotype macrophages.

Here, linker‐free covalent bonding of biomolecule‐based hydrogels to ROS‐functionalized surfaces is demonstrated without the need for initiators and chemical linkers. Gelatin methacryloyl (GelMA) monomers were initially used to react and covalently bond with ROS‐functionalized polymeric surfaces created by atmospheric pressure plasma jet (APPJ) (**Figure** **1**‐i). The roles of monomer molecules, surface charge‐charge interactions, and ionic strength were decoupled by using positively/negatively charged monomers, chitosan and gelatin, at different pH and solvent ionic strengths. To form a robust micro/millimeter‐thick hydrogel layer, an evaporation‐induced enhanced concentration (EIEC) strategy was implemented to increase the number of covalent bonds between hydrogels and anchor sites (Figure 1‐ii). Rehydrated hydrogels showed a strong attachment to ROS‐functionalized polymeric surfaces, as validated by various mechanical tests and comparisons with those formed on conventional silane‐functionalized surfaces. The culture of human mesenchymal stem cells (hMSCs) and M0 phenotype macrophages on the hybrid materials demonstrated low cytotoxicity and minimal immune response. We demonstrated the applicability of this novel strategy in creating HSH constructs with various polymeric surfaces and hydrogels, highlighting its great potential to transform the fabrication of HSH materials with a wide range of modern applications from implantable medical devices to 3D cell culture platforms, soft robotics, and 3D bioprinting.

## 2 Results and Discussion

### 2.1 Covalent Immobilization of GelMA Hydrogel on ROS‐Functionalized Surfaces

**Figure 2:**
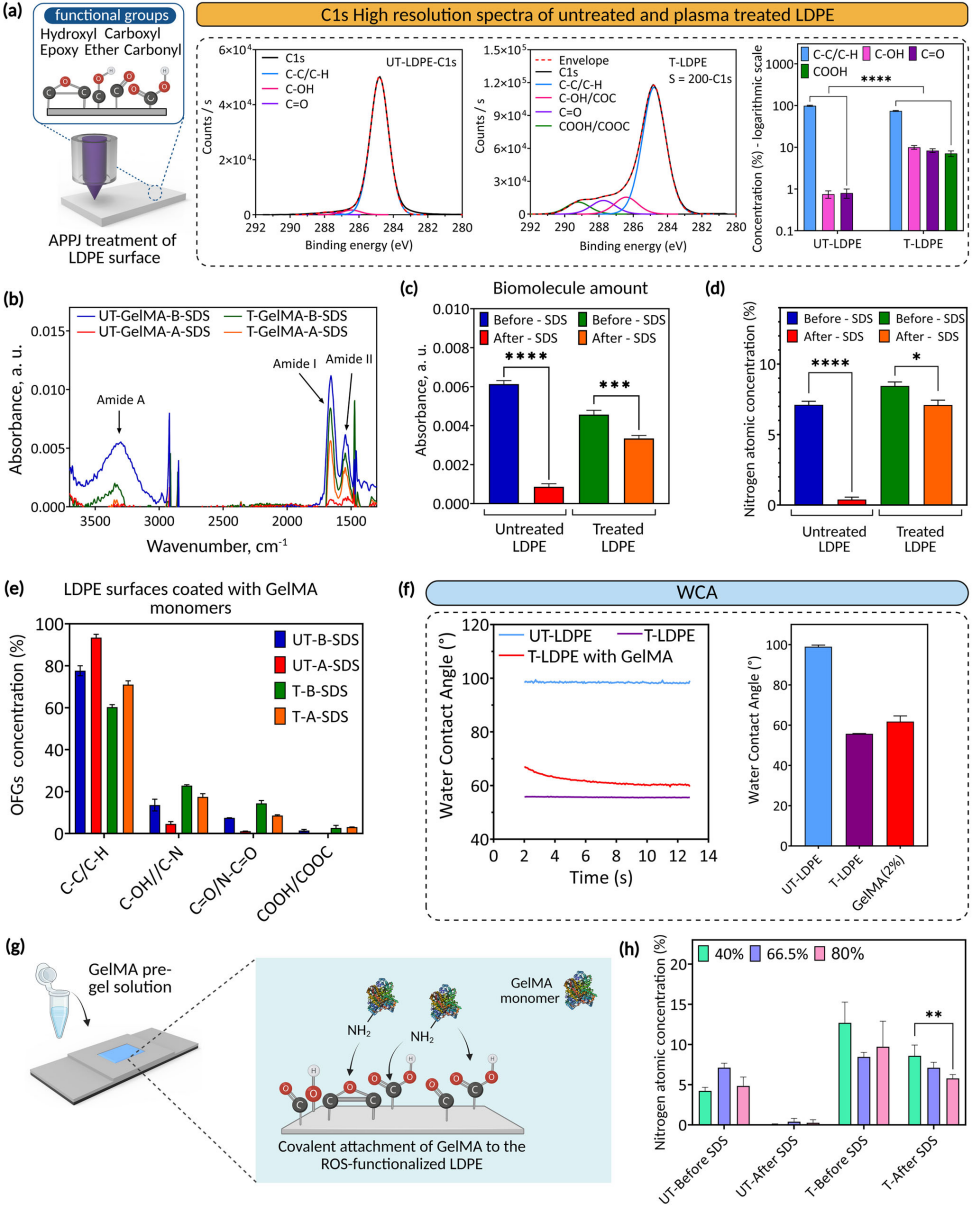
Gelatin methacryloyl (GelMA) is covalently immobilized on ROS‐functionalized (reactive oxygen species‐functionalized) surfaces without the use of chemical linkers. a) atmospheric pressure plasma jet (APPJ) treatment, with a speed of 200 mm min^−1^, leads to the formation of various oxygen‐containing functional groups (OFGs) confirmed by C1s high‐resolution spectra and calculated area percentages of fitted components. Data presented as mean ± SEM, *n* = 3, *p*‐values are calculated using two‐way ANOVA with Tukey correction, ^****^ *p* ≤ 0.0001. b) Attenuated total reflectance Fourier‐transform infrared (ATR‐FTIR) results obtained for untreated (UT) and treated (T) low‐density polyethylene (LDPE) surfaces coated with GelMA monolayer before (B) and after (A) sodium dodecyl sulfate (SDS) washing. The corresponding absorbance peaks for samples with GelMA coatings were subtracted from those without coatings. c) Calculated amide II intensities, as a biomolecule amount, obtained from ATR‐FTIR results. Data presented as mean ± SEM, *n* = 3, *p*‐values are calculated using one‐way ANOVA with Tukey correction, ^****^ *p* ≤ 0.0001, ^***^ *p* ≤ 0.001. d) Calculated nitrogen atm% for untreated and APPJ‐treated LDPE coated with GelMA before and after SDS washing. Data presented as mean ± SEM, *n* = 6, *p*‐values are calculated using one‐way ANOVA with Tukey correction, ^****^ *p* ≤ 0.0001, ^*^ *p* ≤ 0.05. e) The corresponding concentration of molecular composition obtained from C1s high‐resolution spectra from LDPE surfaces coated with GelMA. f) The water contact angle (WCA) was measured as a function of time (from 2 to 12 s) after SDS washing for untreated and APPJ‐treated LDPE with GelMA‐coated samples. g) The possible reaction pathways could be the reaction between amine functional groups in GelMA with carboxyl and epoxide functional groups formed on APPJ‐treated LDPE. h) The effect of the degree of methacrylation (DoM) in GelMA covalent immobilization. Lower DoM results in higher attachment. Data presented as mean ± SEM, *n* = 4, *p*‐values are calculated using two‐way ANOVA with Tukey correction, ^**^ *p* ≤ 0.01).

Polymeric solid substrates were ROS‐functionalized using an atmospheric pressure plasma jet (APPJ) system for the fabrication of HSH constructs. Low‐density polyethylene (LDPE) was selected as a model substrate due to its simple chemical composition. Surfaces were modified using a dynamic treatment approach with a plasma nozzle speed of 200 mm min^−1^, as confirmed by variations in oxygen atomic concentration (atm%) formed on LDPE surfaces (Section S2, Supporting Information). The formation of oxygen‐containing functional groups (OFGs), including C─C/C─H (284.6 eV ± 0.5), C─OH/COC (hydroxyl, ether, and epoxy; 286.5 eV ± 0.5), C═O (carbonyl; 287.8 eV ± 0.5), and COOH/COOR (carboxyl; 289 eV ± 0.5),^[^ ^35^, ^36^, ^37^, ^38^, ^39^, ^40^ ^]^ on LDPE surfaces after APPJ treatment was confirmed by X‐ray photoelectron spectroscopy (XPS) analyses (**Figure** **2**a). While these OFGs include stable and nonreactive species, APPJ treatment also generates ROS, which play a key role in facilitating covalent attachment of biomolecules to the surface, as previously discussed in our earlier work.^[^ ^34^ ^]^ Plasma treatment increased the concentration of C─OH/C─O─C and C═O from ≈1% to 8.5% and 10%, respectively, and also led to the formation of COOH/COOC groups at 7.5%, which were absent on the untreated LDPE. These findings were confirmed by attenuated total reflectance Fourier‐transform infrared (ATR‐FTIR) analyses and water contact angle (WCA) measurements, with the results presented in Section S2 (Supporting Information).

To evaluate the potential of ROS within OFGs, as conjugation sites, generated on LDPE for direct covalent attachment of hydrogels, we placed the APPJ‐treated surfaces in contact with GelMA monomer, serving as a model hydrogel. No initiator was used in this experiment. ATR‐FTIR results obtained from GelMA‐coated samples, with data from uncoated surfaces subtracted, revealed three distinct bands/peaks at 3300, 1650, and 1545 cm^−1^ corresponding to amide A (N─H stretching), amide I (C═O stretching), and amide II (N─H bending and C═O stretching), respectively (Figure 2b). Sodium dodecyl sulfate (SDS), as an ionic detergent, was used to remove physically adsorbed hydrogel monomers from LDPE surfaces. SDS disrupts physical interactions to remove physisorbed biomolecules from substrates while leaving covalent bonds intact.^[^ ^41^ ^]^ The surface‐attached GelMA was quantified based on the intensity of amide II peaks extracted from the ATR‐FTIR spectra (Figure 2c). Upon SDS washing, the absorbance peaks measured on GelMA‐coated untreated surfaces reached background levels, while they remained for the APPJ‐treated samples, confirming the covalent attachment of GelMA to the APPJ‐treated surfaces.

Since APPJ treatment with a speed of 200 mm min^−1^ does not introduce detectable nitrogen‐containing components to the surface chemistry (N atm% < ≈0.3%), measuring nitrogen atm% after GelMA coating can further validate ATR‐FTIR results. XPS survey spectra (Figure S4a, Supporting Information) and the calculated nitrogen atm% (Figure 2d) revealed a significant nitrogen presence of 7.09% ± 0.34% on APPJ‐treated LDPE coated with GelMA after SDS washing. In contrast, only trace nitrogen (0.38% ± 0.17%) remained on untreated samples after SDS washing, reduced from 7.11% ± 0.25% prior to washing. This trace of nitrogen signal likely originates from minor residuals of physically adsorbed GelMA and showed no significant difference compared to untreated LDPE controls (one‐way ANOVA, *p* = 0.5685). Uniform GelMA coverage can also be confirmed by XPS (*n* = 6) and ATR‐FTIR (*n* = 3) results, each obtained at three different surface locations per sample, showing consistent nitrogen atm% and normalized IR peak intensities.

The presence of nitrogen is attributed to the attached GelMA, which contains abundant C─N and N─C═O bonding environments. Curve‐fitted XPS C1s high‐resolution spectra (Figure S4b, Supporting Information) and the area percentage for each fitted component (Figure 2e) showed the presence of carbon‐nitrogen groups, including N─C═O and C─N, on APPJ‐treated LDPE after SDS washing, confirming the covalent immobilization of GelMA monomers to ROS‐functionalized surfaces. In contrast, the near‐zero amount of N─C═O and a significant reduction of C─N on untreated LDPE after SDS washing indicate the successful removal of the physically attached hydrogel. To further confirm covalent attachment, gelatin conjugated with rhodamine B was immobilized on untreated and APPJ‐treated LDPE surfaces and imaged using fluorescence microscopy before and after SDS washing. As shown in Figure S4c (Supporting Information), strong fluorescence signals were detected on APPJ‐treated surfaces coated with gelatin‐rhodamine B after SDS washing, whereas signals were nearly eliminated from untreated controls. The quantified fluorescence intensity (Figure S4d, Supporting Information) further supports these observations.

WCA measurements from GelMA‐coated samples after SDS washing provided further evidence in support of covalent immobilization (Figure 2f). On GelMA‐coated LDPE, the WCA was initially measured at 66° ± 1.61° shortly after the water droplet was placed, then decreased to 61° ± 2.32° and remained stable. In contrast, the WCA on untreated and APPJ‐treated LDPE remained consistent over time with contact angles of 98.9° ± 1.84° and 55.7° ± 2.14°, respectively. The reduction in WCA on GelMA‐coated LDPE is likely due to the variation in surface roughness and absorption of water droplets by immobilized GelMA macromolecules.

To investigate the underlying mechanism of GelMA immobilization on ROS‐functionalized LDPE surfaces, we conducted a targeted surface blocking experiment. APPJ‐treated LDPE surfaces were incubated in 2% (v/v) 2,2,3,4,4,4‐heptafluorobutylamine solution, a fluorinated probe molecule with a primary amine group, to react with reactive OFGs present on APPJ‐treated LDPE. Successful covalent attachment of this molecule was confirmed by XPS analysis before and after SDS washing (Figure S5a, Supporting Information), indicating the coupling of amine groups with OFGs. Among the functional groups identified on the APPJ‐treated LDPE, including COOH, C─OH, C─O─C, and C═O (Figure 2a; Figures S2 and S3, Supporting Information), amine‐epoxide (“click” chemistry) and amine‐carboxyl (amide formation) are chemically feasible under the experimental conditions used in this study (refer to Section S1.5, Supporting Information) (Figure 2g). In contrast, other reaction pathways, such as imine formation with aldehydes or carbonyls, and reactions between amine and hydroxyl or ether groups, are disfavored under such conditions due to either low reactivity or requiring acid catalysis. These mechanisms were, therefore, excluded from consideration.

To determine whether the fluorinated amine probe could occupy and thus block the reactive OFGs on the APPJ‐treated LDPE, we exposed the probe‐treated samples to GelMA (2% w/v) pre‐gel solution. ATR‐FTIR analysis (Figure S5b, Supporting Information) showed a clear reduction in GelMA attachment compared to unblocked controls, confirming that earlier‐reacted functional groups were required to enable GelMA covalent bonding. This observation also implies that other potential GelMA functional groups (such as hydroxyl, carboxyl, or methacrylate side chains) did not play a major role in surface bonding, as no attachment was observed once reactive OFGs (amine‐reactive sites) were blocked. As such, these data suggest the important role of amine‐mediated coupling in GelMA attachment.

We also coated GelMA onto untreated polycaprolactone (PCL), which inherently contains carbonyl (C═O) and ester (O═C─O) groups. After SDS washing, GelMA was removed (Figure S6a, Supporting Information), indicating that such native groups do not facilitate covalent attachment under our experimental conditions. In contrast, APPJ‐treated PCL surfaces showed increased levels of COOH and C─O─C groups (Figure S6b, Supporting Information) and successfully retained GelMA (Figure S6a, Supporting Information). These results further support the conclusion that carboxyl and epoxide functional groups are the primary contributors to covalent GelMA attachment.

During GelMA synthesis, methacrylate groups are grafted onto the primary amine groups of gelatin, reducing the number of available amines that can participate in covalent bonding with reactive OFGs present on APPJ‐treated LDPE surfaces. Therefore, to investigate the effect of the degree of methacrylation (DoM) on covalent immobilization efficiency, GelMA (2% w/v) pre‐gel solutions with different DoM, including 40%, 66.5% (in‐house synthesized), and 80%, were placed in contact with untreated and APPJ‐treated LDPE samples. As shown in Figure 2h, XPS analysis after SDS washing confirmed nitrogen retention under all conditions, indicating successful covalent attachment. However, nitrogen atm% decreased with increasing DoM (80%). This trend can be attributed to the progressive substitution of amine groups by methacrylate moieties during GelMA synthesis, which limits the number of reactive sites available for covalent coupling with carboxyl and/or epoxide groups on APPJ‐treated surfaces. The covalent immobilization of GelMA on ROS‐functionalized surfaces reported here is in agreement with our previous work,^[^ ^34^ ^]^ where various fluorinated carbon brushes with specific functional groups were employed to identify biomolecule reaction pathways on APPJ‐treated solid surfaces.

The robustness of attached hydrogels on solid surfaces depends not only on the formation of covalent bonds but also on the number of bonds formed at the solid‐hydrogel interface. We previously demonstrated that the concentration of peptides covalently attached to plasma‐activated surfaces can be regulated by tuning the peptide solution pH and thus modulating the electric fields at the surface.^[^ ^42^ ^]^ Here, to ensure a maximum number of hydrogel monomers approach the anchorage sites on the APPJ‐treated surfaces for subsequent covalent attachment, a similar strategy was adopted, as discussed in the following section.

#### 2.1.1 Charge‐Charge Interactions Control Covalent Biomolecule Attachment

Surface and monomer charge both influence monomer‐surface interactions.^[^ ^42^ ^]^ Higher interfacial adhesion requires the formation of a high density of covalent bonds at the solid‐hydrogel interface.^[^ ^11^ ^]^ The number of hydrogel monomer molecules arriving on the surface of APPJ‐treated polymers can thus be controlled by regulating the charge interactions at the interface. To investigate this possibility and elucidate the underlying mechanisms, we used chitosan and gelatin as model biomolecules capable of forming hydrogels. The biomolecule solutions were prepared at different pH values to induce specific charges: chitosan at pH 3 to achieve a positively charged molecule enriched with NH_3_⁺, and gelatin at pH 12 as a negatively charged molecule. Another gelatin solution was prepared at pH 7 for comparison; at this pH, gelatin carries a slight positive charge.^[^ ^43^ ^]^

**Figure 3:**
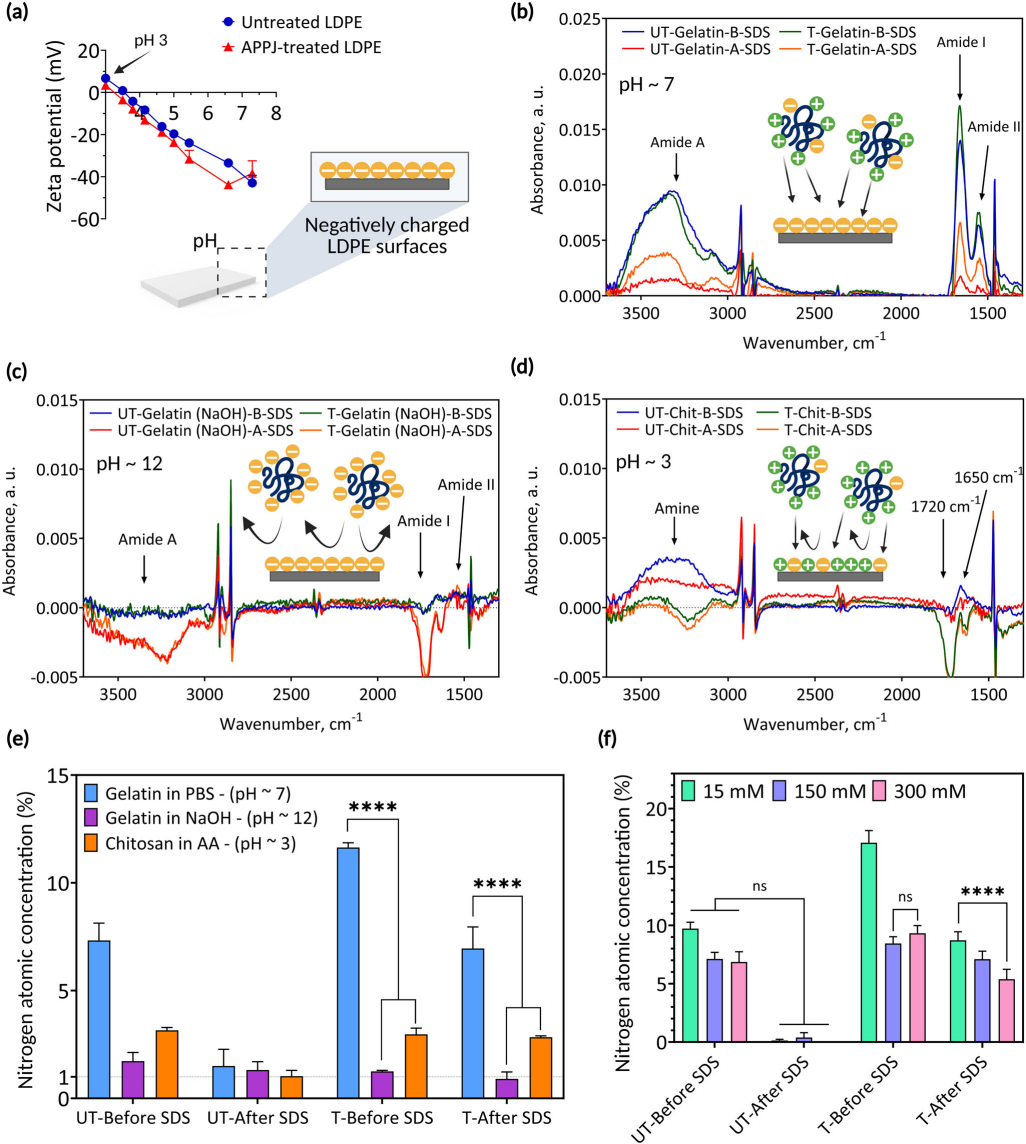
pH controls the monomer‐surface interactions. a) Zeta potential was measured as a function of pH for untreated and atmospheric pressure plasma jet (APPJ)‐treated low‐density polyethylene (LDPE) surfaces. The attenuated total reflectance Fourier‐transform infrared (ATR‐FTIR) results from untreated and APPJ‐treated LDPE surfaces incubated in b) gelatin dissolved in PBS (pH 7), c) gelatin dissolved in NaOH (pH 12), and d) chitosan (chit) dissolved in acetic acid (AA) (pH 3). e) Calculated nitrogen atm% obtained from X‐ray photoelectron spectroscopy (XPS) survey spectra measured for samples coated with gelatin (dissolved in NaOH and PBS) and chitosan (UT: untreated LDPE, T: APPJ‐treated LDPE). Data presented as mean ± SEM, *n* = 3, *p*‐values are calculated using two‐way ANOVA with Tukey correction, ^****^ *p* ≤ 0.0001). f) Effect of ionic strength on covalent immobilization of gelatin methacryloyl (GelMA) on APPJ‐treated LDPE surfaces. Data presented as mean ± SEM, *n* = 3, *p*‐values are calculated using two‐way ANOVA with Tukey correction, ^****^ *p* ≤ 0.0001.

Surface zeta potential measurements for untreated and APPJ‐treated LDPE surfaces (**Figure** **3**a) indicated that both surfaces maintained a negative charge above pH 3. APPJ treatment enhances this negative charge as a result of surface‐attached ROS, including COO^−^ moieties, while untreated surfaces show a negative charge due to photo‐oxidation under ambient conditions.^[^ ^34^, ^44^ ^]^

ATR‐FTIR results (Figure 3b–d) demonstrated significant effects of biomolecule net charges on the concentration of covalently attached biomolecules. Positively charged gelatin at pH 7 exhibited a higher binding affinity to ROS‐functionalized surfaces (Figure 3b), compared to negatively charged gelatin at pH 12 (Figure 3c). The covalent attachment was confirmed by the presence of gelatin on ROS‐functionalized LDPE surfaces incubated at pH 7 after SDS washing (Figure 3b). Despite untreated LDPE being negatively charged, negligible gelatin molecules at pH 7 were observed on untreated LDPE surfaces after SDS washing, excluding electrostatic interaction as the attachment pathway. Negatively charged gelatin at pH 12 did not form covalent bonds and even failed to physically adsorb to either untreated or APPJ‐treated LDPE due to the repulsion between negatively charged gelatin molecules and negatively charged surfaces. Positively charged chitosan at pH 3 was also not detected by ATR‐FTIR on APPJ‐treated surfaces before and after SDS washing (Figure 3d) and it is likely due to the reduced deprotonation of carboxyl groups (COO^−^) present on APPJ‐treated LDPE at pH 3, or to the repulsion between the slightly positively charged surfaces (Figure 3a) and the chitosan monomers.

Nitrogen atm%, representing the concentrations of surface‐attached biomolecules, were calculated from XPS survey spectra and depicted in Figure 3e. This measurement allows us to quantify the surface‐bound biomolecules as no nitrogen‐containing functional groups were detected on the untreated and APPJ‐treated LDPE before immobilization (refer to Figure S2c, Supporting Information). Negligible nitrogen (<1%) was detected on samples incubated in gelatin at pH 12, regardless of APPJ treatment and SDS washing, indicating repulsion between COO^−^ in gelatin and the negatively charged surfaces. Despite chitosan having 15 times more amine functional groups than gelatin molecules,^[^ ^45^ ^]^ it showed lower covalent attachment density on APPJ‐treated LDPE surfaces after SDS washing compared to gelatin at pH 7, indicating a reduced deprotonation of COOH on APPJ‐treated LDPE. Note that the detection of chitosan on LDPE surfaces by XPS, but not ATR‐FTIR, is attributed to the lower sampling depth of XPS (in the range of nanometers) compared to ATR‐FTIR (in the range of micrometers).^[^ ^46^ ^]^ Consequently, deprotonated carboxyl groups on APPJ‐treated LDPE surfaces over the pH range of 3–7 facilitate the approach of positively charged monomers toward the ROS‐functionalized surfaces, where covalent attachment takes place on contact.

To evaluate the influence of electrostatic shielding on covalent biomolecule immobilization, untreated and APPJ‐treated LDPE surfaces were exposed to the GelMA (2% w/v) pre‐gel solution with varying ionic strength at neutral pH. Adjusting the PBS concentration from 15 to 300 millimolar (mM) resulted in a moderate decrease in nitrogen atm% after SDS washing (Figure 3f). However, significant nitrogen content was still retained in all cases, indicating that covalent attachment remains effective under elevated ionic strength conditions. These results together highlight the role of surface charge, biomolecule charge, and solution ionic strength on the number of biomolecules covalently attached to ROS‐functionalized surfaces. Thus, to optimize covalent bonding on the ROS‐functionalized surfaces, careful adjustment of the hydrogel monomer solution's pH and ionic strength is required.

### 2.2 Fabrication of Robustly Attached Micro/Millimeter‐Thick Hydrogel Layers on Solid Surfaces

Monolayers of GelMA hydrogel were shown to be covalently attached to ROS‐functionalized solid surfaces without the need for chemical linkers and initiators, as discussed in Section 2.1. Thicker hydrogel layers can also be formed on ROS‐functionalized surfaces to create HSH constructs. In the case of photo‐crosslinkable hydrogels such as GelMA, monomers can be crosslinked on solid surfaces using photoinitiators and exposure to UV light. As informed by the data presented in Section 2.1.1, adjusting the solution pH to 7 is required to increase the number of monomer molecules arriving on the ROS‐functionalized surfaces and maximize the potential of covalent bond formation at the solid‐hydrogel interface. To enhance the reaction possibility between hydrogel monomers and ROS, ensuring robust anchoring, we implemented an EIEC strategy to maximize the number of covalent bonds at the hydrogel‐solid interface. This strategy is inspired by evaporation‐induced self‐assembly^[^ ^47^ ^]^ and dip‐coating techniques,^[^ ^46^, ^48^, ^49^ ^]^ which involve the deposition of thin films through solvent evaporation. Dehydrated hydrogel coatings can then be rehydrated to form wet hydrogel layers on solid surfaces. To evaluate the adhesion strength of hydrogels coated on the ROS‐functionalized surfaces using the EIEC strategy, we employed three mechanical tests: a peel‐off tape test and a lap shear test for dehydrated conditions, a single‐lap shear test, and a custom “scrape test” for hydrated conditions with the results presented and discussed in the following sections. Note that peel‐off tape tests were only applied to dried GelMA hydrogels, as the tape cannot reliably be applied to rehydrated non‐modified hydrogels due to their soft and non‐adhesive surface. Furthermore, due to their limited cohesive strength, rehydrated GelMA hydrogels are unsuitable for standard peel testing.

#### 2.2.1 Robustness of HSH Constructs in Dehydrated Form

GelMA hydrogel was added to untreated and APPJ‐treated LDPE surfaces and crosslinked using Irgacure and UV light, as detailed in Section S1.15.1 (Supporting Information). The HSH constructs were dried over time to maximize the reaction possibilities between monomers and ROS at the interface.

**Figure 4:**
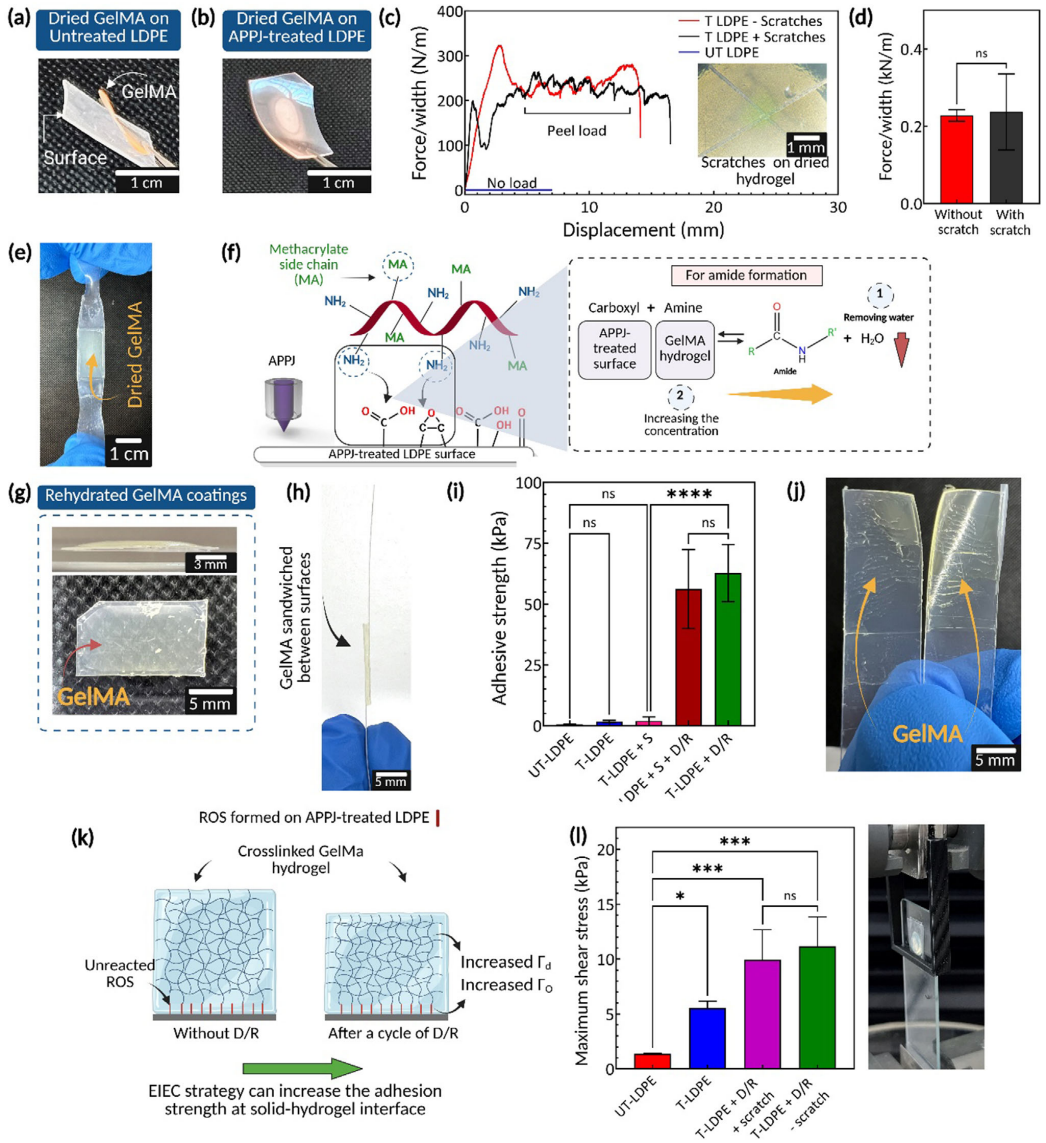
Dehydrated and hydrated gelatin methacryloyl (GelMA) hydrogel strongly attached to ROS‐functionalized (reactive oxygen species‐functionalized) surfaces. a) Dehydrated GelMA hydrogels were spontaneously peeled off from untreated low‐density polyethylene (LDPE) surfaces, while b) they were strongly attached to the atmospheric pressure plasma jet (APPJ)‐treated LDPE surfaces. To visualize GelMA hydrogel, rhodamine B was loaded into the pre‐gel solution prior to the experiment. c) Force/width (N/m) versus displacement for GelMA coated on APPJ‐treated samples with and without scratches. Cross‐shaped scratches were made on one set of samples using a sharp razor blade. To eliminate the effect of sample sizes, the force‐displacement curves were normalized based on the width of each sample. d) The calculated adhesive strength extracted from the plateau region was obtained from the force/width‐displacement curve. Student *t*‐test (*n* = 4) demonstrated no significant difference in adhesion strength between samples with scratches and those without. e) Dried GelMA hydrogel sandwiched between two APPJ‐treated LDPE surfaces after lap shear testing. No delamination was observed, and the LDPE surfaces visibly stretched. f) Amide formation, as one of the reaction pathways, can be facilitated by increasing the monomer concentration of reactants through water evaporation. g) The dried GelMA layer was rehydrated, forming a micro/millimeter‐thick hydrogel layer on APPJ‐treated LDPE. h) GelMA hydrogel sandwiched between two solid surfaces for lap‐shear tests. i) Calculated adhesive strength has confirmed the robustness of GelMA hydrogel and the effectiveness of drying and rehydration (D/R) cycles in optimizing adhesion strength. Data presented as mean ± SEM, *n* = 5, *p*‐values are calculated using one‐way ANOVA with Tukey correction, ^****^ *p* ≤ 0.0001. j) The remaining GelMA on both surfaces demonstrated that failure occurred within the hydrogel matrix. k) A cycle of D/R via the evaporation‐induced enhanced concentration (EIEC) strategy can maximize the covalent bonds per unit area between hydrogel monomers and ROS formed on surfaces and increase the energy dissipation within the hydrogel bulk, thereby increasing the adhesion strength of coatings. l) The calculated maximum stress obtained from scrape tests. A set of dried samples was scratched before rehydration to investigate the effect of surface damage on the adhesion strength of the hydrogel in hydrated form. Data presented as mean ± SEM, *n* = 4, *p*‐values are calculated using one‐way ANOVA with Tukey correction, ^***^ *p* ≤ 0.001, ^*^ *p* ≤ 0.05.

Peel‐off tape tests using standard polystyrene tape strips were conducted to quantify the robustness of dehydrated hydrogels immobilized on LDPE surfaces (Movie S1, Supporting Information). The dried GelMA hydrogel spontaneously peeled off from untreated samples (**Figure** **4**a), preventing any measurement of adhesion with tape tests. The delamination indicates that the intermolecular forces within GelMA molecules are stronger than those between GelMA and untreated surfaces. In contrast, GelMA was still attached to the APPJ‐treated LDPE after the drying process (Figure 4b). To evaluate the adhesion strength of dried GelMA‐coated LDPE, two sets of samples were prepared: one set with cross‐shaped scratches made on the dried hydrogel and another without scratches. The scratches were made to assess whether physical alterations to the hydrogel coatings would compromise their robustness. This type of evaluation is particularly relevant for real‐world biomedical applications such as hydrogel‐coated implantable devices and hydrogel‐based wound dressings, where dehydrated hydrogel coatings may be exposed to mechanical stress or physical surface damage during storage, handling or implantation.^[^ ^50^, ^51^, ^52^ ^]^

The adhesion strength results (Figure 4c) showed consistent adhesion strength up to 220 ± 1 N m^−1^, unaffected by scratches. Force‐displacement curves from the plateau region indicated no significant difference between the results obtained from scratched and unscratched dried hydrogel (Figure 4d), confirming surface damage does not impair hydrogel adhesion. Recent studies on hydrogel adhesion have employed strategies involving custom‐designed chemical linkers, high salt concentrations as crosslinkers, or chemically modified hydrogels to enhance interfacial bonding and achieve substantial adhesion performance under bulk hydrogel testing conditions.^[^ ^53^, ^54^, ^55^ ^]^ In contrast, APPJ‐EIEC strategy offers a single‐step, linker‐free strategy that is applicable to various polymeric surfaces and non‐modified hydrogels, like commercially available GelMA. Importantly, the tape‐based peel tests used in this study provide conservative estimates of adhesion, as the hydrogel coatings remained attached to the substrate after testing, as visually observed and also indicated by the ATR‐FTIR analysis conducted on the samples following the tape test (Figure S7a, Supporting Information).

To further investigate the robustness of dried GelMA coatings, we conducted lap shear tests on dried GelMA hydrogel formed between two APPJ‐treated LDPE substrates (Figure S7b, Supporting Information). The applied force caused deformation of the LDPE substrates, yet the hydrogel remained attached throughout the test with no failure at the GelMA/LDPE interface (Figure 4e; Movies S2 and S3, Supporting Information). This behavior indicates that the coating resisted detachment under mechanical stress. Due to the stretchability of LDPE, which dissipates part of the applied energy during stretching, the resulting force‐displacement values (up to 2000 N m^−1^) reflect both substrate deformation and interfacial adhesion.

Maintaining the adhesion of hydrogel coatings to solid surfaces after multiple drying and rehydration (D/R) cycles is crucial for applications such as diagnostic tools, cell culture platforms, and wound dressing.^[^ ^56^ ^]^ To evaluate the adhesion of dried hydrogel coatings to solid surfaces under such conditions, further testing was conducted involving the hydrogel's solvent evaporation after another cycle of D/R. Briefly, a set of dried GelMA‐coated samples was rehydrated in PBS for up to two months, then dehydrated and stored for five months before conducting tape tests. Remarkably, the adhesion strength remained largely unaffected, indicating the durability of GelMA coatings after one cycle of D/R (Figure S7c, Supporting Information).

The covalent interaction of GelMA monomers with ROS‐functionalized surfaces, as described in Section 2.1, explains the robustness of APPJ‐engineered HSH constructs. The presented results emphasize the potential of ROS to enable stable, long‐lasting attachment of dried macro/millimeter‐thick GelMA layers on APPJ‐treated LDPE surfaces. Considering amide formation as one of the reaction pathways, the continuous evaporation of water increases the concentration of GelMA over time and may facilitate the reaction, as schematically depicted in Figure 4f. Water molecules, as byproducts of amide formation, also hinder close contact between GelMA monomers and ROS. The evaporation serves to increase the collision rate between monomers and various ROS, further enhancing the formation of covalent bonds. Other potential pathways could involve amine‐epoxide click chemistry, which may be facilitated by increased GelMA concentration during solvent evaporation, as well as copolymerization of GelMA with C═C groups presented in ROS.

#### 2.2.2 Robustness of HSH Constructs in Hydrated Form

The covalently attached GelMA hydrogel was shown to adhere strongly to ROS‐functionalized surfaces in dehydrated form. However, for many applications such as hydrogel‐based wound dressings^[^ ^3^ ^]^ and hydrogel‐coated implantable devices,^[^ ^57^ ^]^ it is required that the hydrogel demonstrate strong attachment to the solid substrate in its hydrated state. To evaluate the adhesion strength of hydrogels in their hydrated state as needed for in *vivo* applications, the dried HSH constructs were swelled, forming a thick hydrogel layer (Figure 4g) with a thickness of 0.956 ± 0.093 mm after a cycle of D/R. The bonding strength of the hydrated GelMA hydrogel sandwiched between LDPE surfaces (Figure 4h) was measured using the lap shear test and scrape tests with sample preparation conditions listed in **Table** **1**.

**Table 1:** Sample conditions that were used for the lap shear tests. For each condition, gelatin methacryloyl (GelMA) hydrogel layers were sandwiched between two untreated or atmospheric pressure plasma jet (APPJ)‐treated low‐density polyethylene (LDPE) surfaces. (UT: untreated, T: APPJ‐treated, D/R: drying and rehydration cycle, S: silane functionalized LDPE surfaces using 3‐(Trimethoxysilyl)propyl methacrylate) (TMSPMA)).

Samples name	APPJ treatment	Drying and rehydration	Silane functionalization
UT‐LDPE	−	−	−
T‐LDPE	+	−	−
T‐LDPE + D/R	+	+	−
T‐LDPE + S	+	−	+
T‐LDPE + S + D/R	+	+	+

We compared the adhesion strength of hydrogels attached directly to ROS‐functionalized surfaces after D/R with those attached to silane‐functionalized surfaces using lap shear tests. Silane‐coupling agents such as TMSPMA and APTES, known as bridging molecules, have been widely used to create robust hydrogel coatings.^[^ ^11^, ^58^, ^59^, ^60^ ^]^ The mechanism of reaction between hydrogels and silane agents depends on the two ends of the bridging molecule. One end can hydrolyse and condense with hydroxyl groups presented on surfaces, while another end can covalently bond to hydrogel chains. For TMSPMA used in this study, GelMA hydrogel can copolymerize with the vinyl group in the molecules via a free radical polymerization process,^[^ ^10^ ^]^ and the TMSPMA can be bonded to the hydroxyl groups presented on APPJ‐treated LDPE surface.

The shear stress‐strain curves for all conditions showed a linear increase up to a peak, followed by a load drop (Figure S7d, Supporting Information). Maximum shear stress (Figure 4i) was recorded at 62.73 ± 11 kPa for T‐LDPE + D/R samples, which was significantly higher than 0.48 ± 0.13 and 1.7 ± 0.53 kPa measured for untreated (UT)‐LDPE and APPJ‐treated (T)‐LDPE, respectively. The T‐LDPE + S samples (S: silane functionalized LDPE), which represent the silane coupling method used in Yuk et al.,^[^ ^11^ ^]^ which does not involve D/R steps, showed significantly lower adhesion strength (1.95 ± 1.4 kPa) compared to both the T‐LDPE + S + D/R and T‐LDPE + D/R samples.

These results indicate the strong adhesion of hydrated GelMA to the LDPE surfaces in samples that included the D/R process in their fabrication through the EIEC strategy. To further elucidate the adhesion mechanism, we performed indentation testing on as‐made hydrogel coatings and GelMA coatings after the D/R process, with the results presented in Figure S7e (Supporting Information). Rehydrated samples exhibited higher resistance to indentation, indicating increased stiffness likely caused by partial network densification. In the context of hydrogel adhesion, the interfacial toughness (Γ) can be expressed as the sum of intrinsic interfacial bonding energy (Γ₀) and dissipative energy within the hydrogel bulk (Γ_d_).^[^ ^61^ ^]^ Hydrogel network densification during drying can introduce reversible interactions such as hydrogen bonding or crystalline regions, which can enhance energy dissipation during deformation.^[^ ^62^, ^63^, ^64^ ^]^ Consistent with these findings, lap shear tests in our study showed significantly enhanced adhesion in D/R samples, with cohesive failure observed within the hydrogel (Figure 4j). These results indicate that the improved adhesion can be due to a combined effect of increased Γ₀, via covalent bonding facilitated by the EIEC process and enhanced Γ_d_ through physical network reinforcement, as schematically depicted in Figure 4k. Note that to minimize drying of hydrogels under ambient conditions, all the samples were tested within a maximum of 10 s after removal from PBS (Movie S4, Supporting Information).

The adhesion strength of GelMA hydrogel coated on ROS‐functionalized surfaces via EIEC strategy is significantly higher than that achieved on silane‐functionalized surfaces, a method previously reported by Yuk et al.,^[^ ^11^ ^]^ who achieved robust attachment of chemically modified photo‐crosslinkable hydrogels to TMSPMA‐coated nonpolymeric surfaces functionalized by vacuum oxygen plasma. Despite the strong adhesion strength reported in their work, it proved inapplicable to ROS‐functionalized polymeric surfaces and nonchemically modified hydrogels. The APPJ‐EIEC strategy not only improved the adhesion of GelMA coated on TMSPMA‐functionalized surfaces but also facilitated comparable adhesion strength for GelMA directly formed on ROS‐functionalized surfaces without the use of chemical linkers. Additionally, silane coating of samples was shown to decrease wettability, as evidenced by the increase in WCA from 55.7° ± 2.14° for APPJ‐treated to 73.64° ± 6.7° for silane‐coated samples (Figure S7f, Supporting Information). The nonpolarity of C═C groups in TMSPMA molecules causes a reduction in wettability, which can be a significant drawback since it prevents the hydrogel monomer droplets from fully spreading across the samples. Rehydrated HSH constructs, which were made using the APPJ‐EIEC strategy, have remarkable adhesion comparable to previous studies while eliminating the need for multi‐step wet chemistry. Achieving similar adhesion levels often requires fabricating adhesive or tough hydrogels at high monomer concentrations^[^ ^65^ ^]^ or the utilization of various chemical linkers.^[^ ^66^, ^67^, ^68^, ^69^, ^70^, ^71^, ^72^ ^]^

To assess the integrity of the HSH constructs against surface damage that might occur in the dried form, cross‐shape scratches were applied to a set of samples, labeled T‐LDPE + D/R + scratches. Hydrogel coatings must resist delamination during rehydration, even with surface damage. A scrape test was conducted to measure the adhesion strength of rehydrated GelMA hydrogel on LDPE surfaces and evaluate the impact of scratches.

Stress versus displacement curves (Figure S7g, Supporting Information) and calculated maximum shear stress (Figure 4l) showed a significant difference in the adhesion strength between GelMA hydrogel attached to UT‐LDPE and T‐LDPE samples, with values measured at 1.3 ± 0.04 and 5.5 ± 0.65 kPa, respectively. APPJ treatment notably increased bonding strength fourfold compared to UT‐LDPE. The GelMA hydrogel demonstrated superior bonding strength to T‐LDPE + D/R samples, achieving the highest ultimate shear stress up to 9.9 ± 2.7 and 11.1 ± 2.6 kPa with and without scratches, respectively. One‐way ANOVA tests indicated no significant difference in maximum shear stress between T‐LDPE + D/R with and without scratches, indicating that surface damage did not affect the adhesion in their hydrated forms. These findings further support the strong adhesion observed in the dried state (Figure 4c,d), where covalently attached GelMA on ROS‐functionalized surfaces remained intact despite surface scratches.

The consistent adhesion strength, even after physical alterations, highlights the durability of the hydrogel coatings under both dried and hydrated conditions. Such reliable adhesion is crucial for various biomedical applications where hydrogel coatings must withstand mechanical stress. The HSH constructs achieved via the APPJ‐EIEC strategy have been shown that they have the capability to be stored in dried forms for up to months and rehydrated once needed. The following section measures the water stability and swelling ratio of these hybrid constructs.

### 2.3 Static versus Dynamic Water Stability and Swelling Behavior

The stability of hydrogel coatings on solid surfaces in aqueous environments is crucial for applications such as implantable devices,^[^ ^60^, ^73^, ^74^ ^]^ and 3D cell culture.^[^ ^75^, ^76^ ^]^ To evaluate the durability of hydrogel coatings in aqueous environments, water stability tests were carried out in static and dynamic configurations.

**Figure 5:**
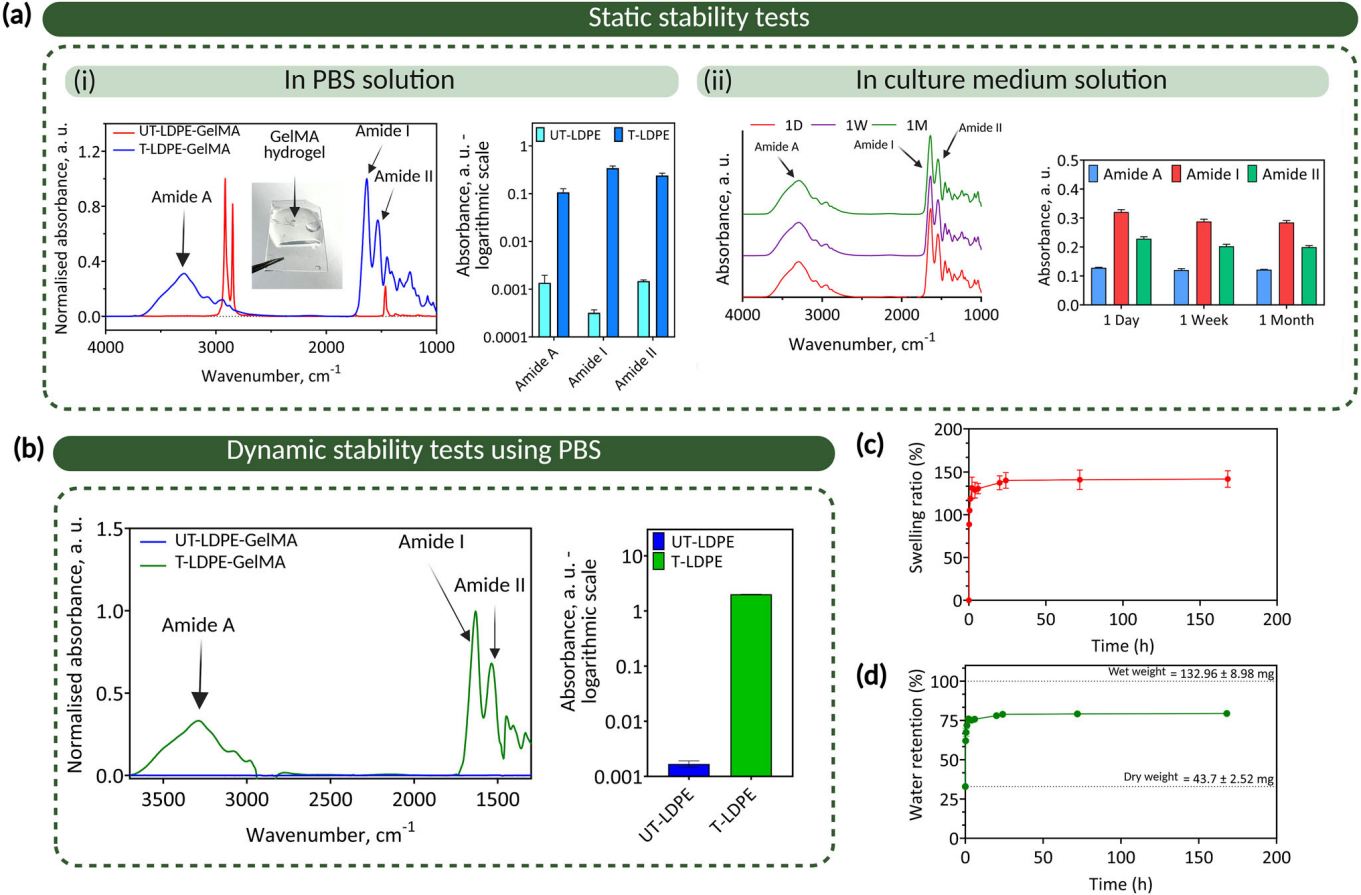
Hydrogel coatings demonstrate high stability in aqueous environments. Static and dynamic water stability tests demonstrated the capability of atmospheric pressure plasma jet (APPJ)‐treated surfaces to provide conjugation sites for covalent bonding of gelatin methacryloyl (GelMA) hydrogel as durable coatings. a) Static stability tests of GelMA‐coated low‐density polyethylene (LDPE) in (i) PBS and (ii) cell culture medium. For both cases, attenuated total reflectance Fourier‐transform infrared (ATR‐FTIR) spectra and corresponding absorbance intensities for amide peaks indicate the presence of GelMA on the APPJ‐treated LDPE surfaces (T‐LDPE) after long‐term incubation. (For samples incubated in cell culture medium 1D: 1 day, 1 W: 1 week, and 1M: 1 month). b) For the dynamic method, ATR‐FTIR results from APPJ‐treated and untreated LDPE were subtracted from bare LDPE, and the sum of the normalized amide peaks intensities confirmed the presence of GelMA hydrogel on the APPJ‐treated LDPE. c) Swelling ratio of dried hydrogel as a function of incubation time in PBS for up to 1 week. d) Water retention calculation of dried hydrogels as a function of time incubated in PBS up to 1 week. The swelled hydrogel reaches ≈79% of its original weight in wet conditions. All data presented as mean ± SEM, *n* = 3.

In the static configuration, the samples were incubated in PBS at 37 °C to monitor stability over a period of up to two months. ATR‐FTIR results showed the presence of GelMA hydrogel on APPJ‐treated samples even after a 2‐month incubation in PBS (**Figure** **5**a‐i; Figure S8, Supporting Information). To further assess the long‐term stability of the covalently bonded hydrogel coatings under physiologically relevant conditions, HSH constructs were incubated in culture medium (RPMI 1640) at 37 °C for one month. ATR‐FTIR analysis confirmed the continued presence of GelMA hydrogel on APPJ‐treated LDPE surfaces after one month (Figure 5a‐ii), consistent with results obtained from those incubated in PBS. In the dynamic configuration, samples were subjected to PBS flow at a rate of 50 mL min^−1^ to examine their stability over a time frame of 12 h. Figure 5b shows the summation of normalized intensities corresponding to the amide I, II peaks, and amide A bands from subtracted ATR‐FTIR results for untreated and APPJ‐treated samples. On untreated surfaces, the intensity of the amide protein peaks associated with GelMA reaches the background level, clearly highlighting the role of ROS in improving hydrogel adhesion.

These results collectively indicate the robustness of the hydrogel coating attached to the ROS‐functionalized solid substrate under both static and dynamic wet conditions.

Hydrogels can be rehydrated when in contact with body fluids or prior to usage using specific media containing bioactive reagents of choice.^[^ ^77^ ^]^ Thus, swelling tests were also conducted to evaluate the hydrogel's capacity to retain water molecules in comparison to the original wet and dry states.

To investigate the swelling ratio and determine the final rehydrated weight of GelMA‐coated surfaces compared to their dry and wet conditions, the samples were first dried and subsequently immersed in PBS for up to a week. As shown in Figure 5c, dried GelMA‐coated LDPE samples swelled up to 141 ± 9.65% of their dried form. Due to the delamination of dried GelMA hydrogels from untreated LDPE, this test was not applicable to untreated samples. Water retention capacity was calculated based on the original weight of the HSH construct in the wet condition, as detailed in Section S1.13 (Supporting Information). Figure 5d shows that rehydrated samples reached ≈79.5% of their original weight when wet due to the denser network, validating the results obtained from indentation tests (Figure S7e, Supporting Information). During evaporation, GelMA monomers were covalently bonded to the ROS anchorage sites at the solid‐hydrogel interface, leading to a 20% reduction in capacity to retain water compared to the original hydrated weight. These findings further validate the results obtained from the lap‐shear and scrape mechanical tests (Figure 4i,l), indicating higher GelMA adhesion to the APPJ‐treated surfaces.

### 2.4 hMSCs Viability and Adhesion Assessment on Hybrid HSH Constructs

To assess the biocompatibility of APPJ‐engineered HSH materials, cell viability, proliferation, and adhesion behavior were evaluated using hMSCs cultured on both untreated and APPJ‐treated LDPE surfaces with GelMA coatings. hMSCs have been widely used in tissue engineering due to their multipotency and critical role in regenerative medicine.^[^ ^78^ ^]^ The cytotoxicity of the hybrid constructs was evaluated by measuring the metabolic activity of hMSCs (WST‐8/CCK kit) and performing DNA counting.

**Figure 6:**
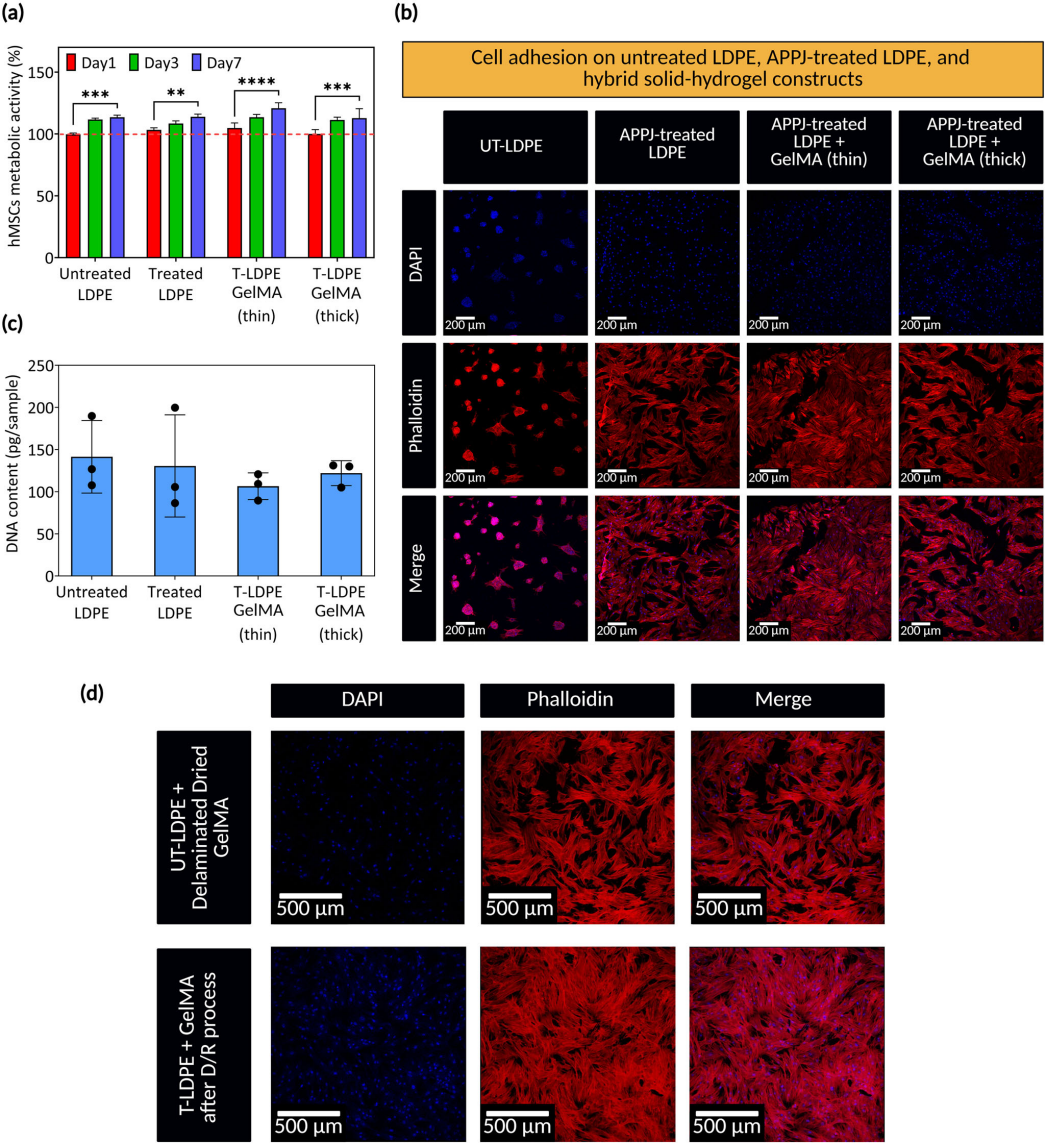
Hybrid solid‐hydrogel (HSH) constructs support human mesenchymal stem cells (hMSCs) viability, adhesion, and proliferation. a) The viability results of hMSCs seeded on untreated low‐density polyethylene (UT‐LDPE) and atmospheric pressure plasma jet (APPJ)‐treated LDPE (T‐LDPE) surfaces and those samples with hydrogel coatings. The cell counting kit utilized here works based on metabolic activity. b) Cell adhesion results provided by confocal imaging of Alexa FluorTM 488 and DAPI‐stained cells show the cytoskeleton (actins) and nuclei (nucleic acids) of the cells in red and blue, respectively. Merged images demonstrate the spindle‐like shape of cells adhered to APPJ‐treated samples and those with gelatin methacryloyl (GelMA) coating. c) DNA quantification of each sample indicates no significant difference in the number of attached cells. d) Cell adhesion results obtained from UT‐LDPE after drying GelMA and T‐LDPE after a cycle of D/R. Note that dried GelMA was delaminated from UT‐LDPE. Data presented as mean ± SEM, *n* = 3, *p*‐values are calculated using two‐way ANOVA with Tukey correction, ^****^ *p* ≤ 0.0001, ^***^ *p* ≤ 0.001, ^**^ *p* ≤ 0.01.

The metabolic activity of hMSCs, as depicted in **Figure** **6**a, increased over the 7‐day period across all samples, including untreated LDPE, APPJ‐treated LDPE, and those with GelMA coatings. The upward trend in metabolic activity suggests that the HSH constructs are biocompatible and effectively support cell proliferation. DNA quantification on day 7 (Figure S9a, Supporting Information) mirrored the metabolic activity results, further confirming cell viability. To assess whether the D/R process impacts cell viability and proliferation, the metabolic activity of hMSCs was evaluated on rehydrated samples. The results showed an increase in metabolic activity in all experimental groups, confirming that the biocompatibility of the hybrid constructs remained unchanged (Figure S9b,c, Supporting Information).

Cytoskeleton staining after one day of culturing revealed distinct differences in cell morphology and distribution across the various samples (Figure 6b). On bare LDPE, hMSCs exhibited a less uniform distribution, displaying a rounded morphology, indicating reduced cell attachment and spreading. In contrast, APPJ‐treated LDPE surfaces and those with GelMA coatings showed a significant improvement, with hMSCs adopting an expanded spindle‐like shape, indicative of a more elongated and migratory phenotype. DNA extraction and measurement using the PicoGreen assay on the same samples (Figure 6c) confirmed no significant difference in the number of attached cells across all samples. The results indicated that while surface chemistry and GelMA coatings influence cell morphology and distribution, they do not necessarily affect the overall number of cells that initially adhered to the surfaces. The D/R cycles did not affect cellular morphology, as shown in Figure 6d. Cell distribution was more homogeneous on APPJ‐treated samples with rehydrated GelMA than in untreated samples. Since GelMA hydrogels delaminated from untreated LDPE after drying, the physically attached GelMA hydrogels to untreated LDPE led to cells spreading across the untreated LDPE as well. Compared to Figure 6b, cells spread more uniformly after D/R cycles. This improved homogeneity in cell distribution could be attributed to the swelling of the GelMA hydrogel in the culture media during rehydration, which may promote protein adsorption and, consequently, cell adhesion and spreading behavior.

Surface chemistry plays a crucial role in influencing adhesion tendencies and subsequent intracellular signaling pathways.^[^ ^79^, ^80^, ^81^ ^]^ Increased hydrophilicity of APPJ‐treated LDPE enhances cell adhesion and spreading compared to bare LDPE.^[^ ^82^ ^]^ Cells grown on untreated LDPE exhibited a round and less spread morphology, likely due to hydrophobicity, leading to nonhomogeneous adhesion. This uneven adhesion affects cell‐cell interactions, signaling the cells to maintain a more compact and spherical shape, rather than spreading out. Similar observations were reported by Vito et al., where hMSCs maintained a rounded shape on bare LDPE surfaces.^[^ ^83^ ^]^ Comparable behavior was observed in other cell lines, such as fibroblast and endothelial cells, which spread on plasma‐treated LDPE but remained rounded on untreated samples.^[^ ^84^, ^85^ ^]^ The presence of GelMA on APPJ‐treated LDPE further improves cell adhesion and spreading due to its superior biological properties, which promote the cell‐substrate interaction. Taken together, these results confirm the biocompatibility of APPJ‐engineered HSH constructs.

### 2.5 Macrophage Viability and Stimulation Assessment

The implantation of foreign materials into the body or in contact with open wounds can potentially trigger adverse immune reactions, causing chronic inflammation, impairing healing, or leading to material rejection.^[^ ^86^ ^]^ The functionality of biomaterial devices can be compromised by foreign body reaction, which hinders desired interactions between the biomaterial and host tissue.^[^ ^87^ ^]^ Upon implantation, macrophages are recruited to the biomaterial site, where they play a pivotal role in the host's immune responses.^[^ ^88^ ^]^ This recruitment and macrophage activity are critical not only for the initial immune reaction but also for the long‐term integration of biomaterials. To understand how macrophages respond to the APPJ‐engineered HSH constructs, we investigated macrophage viability and activation upon contact with these constructs.

**Figure 7:**
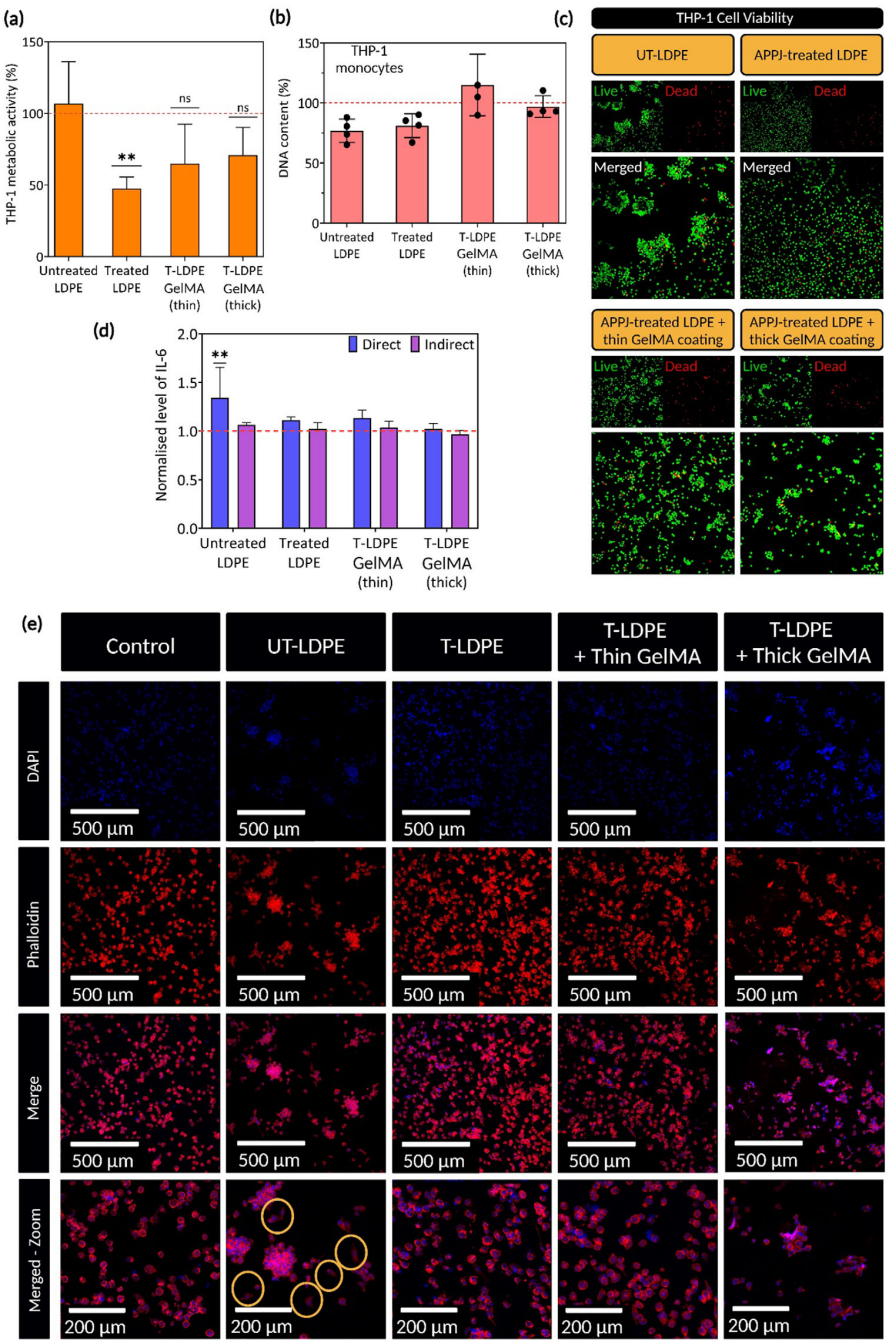
Hybrid solid‐hydrogel (HSH) constructs demonstrate no sign of adverse immune reactions. a) Metabolic activity of THP‐1 derived macrophages cultured on each sample. Data presented as mean ± SEM, *n* = 4, *p*‐values are calculated using one‐way ANOVA with Dunnett correction, ^**^ *p* ≤ 0.01. b) DNA counting obtained from cultured macrophages shows the viability and the quantity of attached macrophages on each sample. c) Live‐dead results provided by confocal imaging stained THP‐1 derived macrophages (live cells in green and dead cells in red), which confirmed the cytocompatibility of untreated low‐density polyethylene (UT‐LDPE) and atmospheric pressure plasma jet (APPJ)‐treated LDPE (T‐LDPE) with and without gelatin methacryloyl (GelMA) coatings. d) The intensity of secreted IL‐6 by macrophages was normalized to the control (nonstimulated cells cultured on the well plate), and e) Confocal images of Alexa FluorTM 488 Phalloidin (cytoskeleton, red) and DAPI (nuclei, blue) stained cells indicating the morphology and distribution of cells. Merged zoom image of cells on UT‐LDPE confirms a pro‐inflammatory response compared to the control, following the smaller and more elongated morphology (yellow circles). Data presented as mean ± SEM, *n* = 4, *p*‐values are calculated using two‐way ANOVA with Dunnett correction, ^**^ *p* ≤ 0.01.

**Figure** **7**a shows the metabolic activity of THP‐1 derived macrophages cultured on each sample. The results indicated higher metabolic activity of macrophages attached to untreated LDPE surfaces compared to APPJ‐treated samples and those with hydrogel coatings. However, statistical analysis showed no significant difference in metabolic activity between samples and controls. The variation in metabolic activity of macrophages can be affected by two factors of cell viability and immune responses upon contact with foreign objects.^[^ ^89^ ^]^ To investigate whether the observed changes in metabolic activity were due to cell death or an immune response, complementary DNA counting and live‐dead assays were performed.

Despite the trends observed in metabolic activity, DNA counting results, shown in Figure 7b, revealed no significant differences in the number of attached cells across the different sample conditions with controls, suggesting that cell viability remains consistent. Live‐dead assays further confirmed the results obtained from DNA counting. The results presented in Figure 7c showed no significant presence of dead cells (red objects) on either untreated or APPJ‐treated surfaces, including those with GelMA coatings. These findings confirm that the lower metabolic activity observed in APPJ‐treated LDPE and those with hydrogel coatings, and the higher metabolic activity observed in APPJ‐treated LDPE are not due to decreased cell viability but could be influenced by other factors, such as immune responses.

To investigate pro‐inflammatory responses, interleukin 6 (IL‐6) cytokine secretion was quantified using both direct and indirect methods, as described in Section S1.19 (Supporting Information), and the results are depicted in Figure 7d. The results show that when cells were cultured directly on the samples, untreated LDPE surfaces induced increased IL‐6 secretion, despite being nontoxic. In contrast, the indirect method showed no inflammatory responses, indicating that the potential release of non‐crosslinked GelMA monomers or the presence of Irgacure in the GelMA did not stimulate macrophages. The overall findings confirmed the biocompatibility of the HSH constructs, particularly in avoiding immune responses.

Figure 7e illustrates the morphology of THP‐1 derived microphages in contact with the samples. Untreated LDPE surfaces were shown to trigger pro‐inflammatory responses, as indicated by the presence of smaller macrophages and less rounded cells, transitioning to the M1 phenotype. These observations are in agreement with the results reviewed by Marie‐Claire et al.^[^ ^90^ ^]^ The hydrophobic nature of untreated LDPE may contribute to reduced cell adhesion and increased aggregation. In contrast, APPJ‐treated LDPE surfaces, which are more hydrophilic due to the surface‐attached OFGs, displayed a more homogenous dispersion of macrophages. This result confirmed that surface modifications can significantly influence cell adhesion and functionality. When comparing APPJ‐treated surfaces with thin and thick GelMA coatings, a more uniform macrophage distribution was observed on samples with thin GelMA coatings. The thicker GelMA coatings, however, exhibited more cell aggregation. This aggregation could be attributed to variations in hydrogel thickness and roughness, as well as potential partial GelMA degradation leading to cell detachment over time. The pattern of aggregation observed in the thick coatings is consistent with the live‐dead assay results (Figure 7c).

The results presented in this section validate the cytocompatibility of APPJ‐treated surfaces and those coated with GelMA hydrogels. High viability and normal morphology of THP‐1 derived macrophages were observed on HSH structures, with no indications of adverse immune reactions.

### 2.6 Broad Applicability of Linker‐Free Covalent Bonding Between Diverse Polymeric Substrates and Hydrogels Using APPJ‐EIEC Strategy

To demonstrate that the linker‐free fabrication method of HSH constructs, using APPJ surface functionalization and EIEC strategy, is not specific to only GelMA and LDPE, we applied this approach to different polymeric substrates and hydrogels.

PCL and polytetrafluoroethylene (PTFE), two widely used polymers in biomedical applications, were selected due to their distinct chemical compositions. PCL, with its OFGs, and PTFE, enriched with carbon‐fluorine bonds, were subjected to APPJ treatment to assess the polymer‐independent ROS formation, as conjugation sites, at the plasma‐polymer interface. XPS survey spectra (Figure S10a, Supporting Information) confirmed OFGs formation on PCL and PTFE surfaces, with oxygen atm% increasing from 23.3% ± 0.72 to 29.5% ± 1.88 for PCL and from less than 0.1% to 1.9% ± 0.17 for PTFE after APPJ treatment.

Variations in oxygen atm% formed on LDPE (discussed in Section S2, Supporting Information), PCL, and PTFE substrates can be attributed to differences in bond dissociation energies (BDEs). LDPE, with its relatively low BDEs for C─C and C─H bonds (≈250–300 and ≈337–420 kJ mol^−1^, respectively^[^ ^91^ ^]^), experiences more pronounced bond cleavage during plasma treatment compared to PCL and PTFE. This process leads to a higher formation of reactive carbon‐based intermediates, which readily interact with oxygen species in the plasma,^[^ ^46^ ^]^ resulting in the significant formation of OFGs on LDPE surfaces (Figure S2b,c, Supporting Information). These differences in OFGs formation highlight how the molecular structures and bond stability of each polymer directly influence the extent of ROS incorporation as a result of APPJ activation.

**Figure 8:**
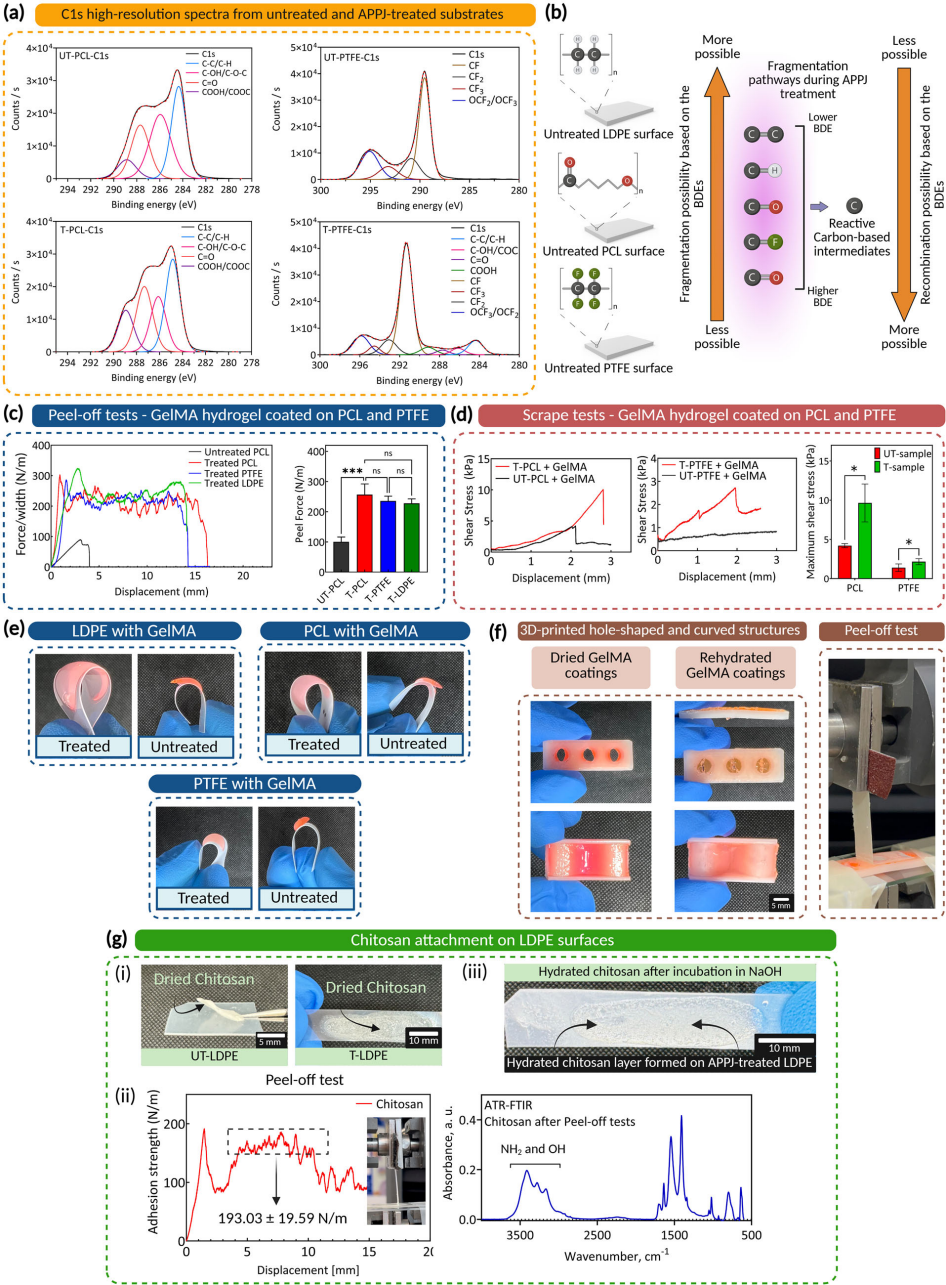
Atmospheric pressure plasma jet‐evaporation‐induced enhanced concentration (APPJ‐EIEC) strategy for fabricating hybrid solid‐hydrogel (HSH) constructs is applicable to a wide range of polymeric substrates and hydrogels. a) C1s high‐resolution spectra of untreated (UT) and APPJ‐treated (T) polycaprolactone (PCL) and polytetrafluoroethylene (PTFE) revealed the chemical composition of functional groups on these surfaces before and after APPJ treatment. b) Schematic illustration of surface chemical changes, based on bond dissociation energies (BDEs), in low‐density polyethylene (LDPE), PCL, and PTFE as a result of APPJ treatment. c) Adhesion strength of dried hydrogel coated on PCL and PTFE obtained by peel‐off tape tests. Calculated peel force from the plateau region has demonstrated the strong adhesion of hydrogels to ROS‐functionalized (reactive oxygen species‐functionalized) PCL and PTFE. Data presented as mean ± SEM, *n* = 4, *p*‐values are calculated using one‐way ANOVA with Bonferroni correction, ^**^ *p* ≤ 0.01. d) Scrape tests were conducted to evaluate the adhesion strength of gelatin methacryloyl (GelMA) hydrogel on PCL and PTFE in hydrated form. Data presented as mean ± SEM, *n* = 4, *p*‐values are calculated using *t*‐tests, ^*^ *p* ≤ 0.05. e) GelMA hydrogel coated on LDPE, PCL, and PTFE surfaces after being removed from PBS. Bending forces led to the delamination of the coating from untreated samples, while the coatings adhered to APPJ‐treated samples after bending forces were applied. f) APPJ‐EIEC strategy can also be applied to non‐flat surfaces. 3D‐printed PCL structures, including a bone‐plate shape and a curved shape, were used as substrates. Peel‐off tests were performed on samples, and the dried hydrogel remained on the surfaces after testing. Dried hydrogels were then rehydrated, and a micro/millimeter‐thick hydrogel layer was formed on these structures without delamination. g) (i) The dried chitosan layer was peeled off from the untreated LDPE while they were attached to the APPJ‐treated LDPE. (ii) Peel‐off tests were performed to evaluate the adhesion strength of chitosan to APPJ‐treated LDPE in dehydrated forms (*n* = 4). (iii) Dried chitosan can be rehydrated in a basic solution to be both swelled and crosslinked.

The C1s high‐resolution spectra for PCL and PTFE, both before and after APPJ treatment, were obtained from XPS measurements and are presented in **Figure** **8**a, with the quantified concentrations shown in Figure S10b (Supporting Information). Compared to LDPE, PCL and PTFE have higher BDEs for their respective chemical bonds. PCL contains C─C, C─H, C─O (BDE of ≈350–400 kJ mol^−1^), and C═O (BDE of 749─800 kJ mol^−1[^ ^91^ ^]^) bonds. While the C─C and C─H bonds can fragment, forming reactive carbon‐based intermediates, the overall degree of bond breakage is less extensive compared to LDPE, due to the higher energy required to break C─O and C═O. Since PCL already includes oxygen‐containing groups, fragmentation and recombination with oxygen species during APPJ treatment lead to the reformation of similar OFGs. This behavior results in a noticeable, but moderate, increase in oxygen content in the PCL compared to LDPE after APPJ treatment. PTFE, on the other hand, primarily contains C─C and C─F (BDE of ≈490–536 kJ mol^−1[^ ^91^ ^]^) bonds.^[^ ^38^ ^]^ Due to the higher BDEs of C─F bonds, they are less likely to break during APPJ treatment. Even if fragmentation occurs, the reformation of C─F bonds is thermodynamically favored over C─O bonds. Consequently, the overall incorporation of oxygen species is limited, leading to lower OFGs formation compared to PCL and LDPE. Figure 8b schematically illustrates the chemical compositions of untreated PCL, PTFE, and LDPE, and the pathways for bond fragmentation and recombination processes during APPJ treatment.

The results obtained from XPS were further validated by ATR‐FTIR (Figure S10c, Supporting Information) and WCA measurements (Figure S10d, Supporting Information) and support the formation of OFGs across different polymers with varying chemical compositions. These findings highlight the substrate‐independent nature of the APPJ treatment, making it a versatile approach for preparing diverse polymeric surfaces for hydrogel attachment.

The hydrogel attachment to ROS‐functionalized PCL and PTFE was further evaluated to assess the robustness of the HSH constructs under both dehydrated and hydrated conditions. As explained in Section 2.2, the covalent bonding between ROS and GelMA monomers is crucial for achieving strong adhesion. The robustness and interfacial adhesion strength of GelMA coatings on PCL and PTFE surfaces were measured using peel‐off and scrape tests. Figure 8c illustrates the normalized adhesion strength curve and the corresponding quantified peel load. The adhesion strength of dried GelMA coated on PCL and PTFE was comparable to that on LDPE, highlighting the efficacy of ROS formed on PCL and PTFE substrates. While dried GelMA hydrogel spontaneously delaminated from untreated PTFE, it remained adhered to untreated PCL before peel‐off tests. However, the force required to detach dried GelMA from untreated PCL was significantly lower than that of APPJ‐treated PCL. Note that the dried GelMA hydrogels remained adhered to all APPJ‐treated polymeric surfaces after peel‐off tests. The adhesion strength observed is comparable to our previous study, where acrylamide hydrogels were covalently bonded to radical‐functionalized surfaces activated via a low‐pressure PIII process.^[^ ^12^ ^]^ Further evidence of the strong attachment of GelMA to APPJ‐treated PCL and PTFE was provided by the shear stress‐strain graphs from the scrape tests in hydrated forms (Figure 8d). GelMA hydrogel remained adhered to both untreated and treated PCL, with a higher force required for removal from APPJ‐treated samples, showing an adhesion strength increase from 4.1 to ≈9.7 kPa. On untreated PTFE, GelMA hydrogel showed significant mobility, indicating minimal adhesion, with an adhesion strength of 0.7 kPa compared to 2.1 kPa for APPJ‐treated PTFE.

The APPJ‐EIEC strategy enables linker‐free covalent bonding, resulting in strong adhesion of hydrogels to different APPJ‐treated polymeric surfaces. This robust attachment was demonstrated by the rehydrated GelMA hydrogels, which remained firmly adhered to LDPE, PCL and superhydrophobic PTFE surfaces, with the latter exhibiting a WCA of 117° ± 1.32 (Figure S10d and Movies S5–S7, Supporting Information). Despite rigorous manual testing, including torsion and bending, the hydrogels maintained their adhesion to ROS‐functionalized polymeric surfaces. However, they delaminated from untreated surfaces after bending, with varying forces required for delamination. GelMA hydrogel delaminated from untreated PTFE after a slight bending, while it required more bending stress to delaminate from PCL and LDPE samples (Figure 8e). The APPJ‐EIEC strategy to create hydrogel coatings is also effective on curved and hole‐shaped structures, as depicted in Figure 8f. The dried GelMA remained intact on these surfaces post‐peel‐off tests (Movies S8 and S9, Supporting Information), and the rehydrated GelMA continued to adhere to the samples after removal from PBS, as shown in Movie S10 (Supporting Information).

The efficacy of the linker‐free APPJ‐EIEC strategy was further validated using chitosan as a chemically crosslinkable hydrogel model. Chitosan, a naturally derived hydrogel, has been widely used in tissue engineering due to its remarkable antibacterial properties.^[^ ^92^, ^93^ ^]^ The monomers of chemically crosslinkable hydrogels can be initially bound to APPJ‐treated substrates, after which they can be crosslinked upon rehydration in their corresponding crosslinkers. Informed by the results on the role of charge‐charge interactions in regulating the number of covalent bonds between monomers and ROS (Figure 3), we initially dissolved chitosan in acetic acid at a pH of 3, then adjusted it to pH 7 with NaOH. At the neutral pH, deprotonated carboxyl groups in ROS can attract the protonated amine groups in chitosan, facilitating covalent attachment. Chitosan monomers were then applied to untreated and APPJ‐treated LDPE surfaces, followed by a drying process. Upon dehydration, chitosan was delaminated from untreated LDPE but adhered to the APPJ‐treated surfaces (Figure 8g‐i; Movie S11, Supporting Information). The delamination from untreated LDPE highlighted the absence of covalent binding sites, while the presence of thick chitosan monomers on APPJ‐treated LDPE surfaces was due to covalent bonding. The possible mechanism of covalent attachment may involve the reaction between the abundant amine groups in chitosan and carboxyl and epoxy functional groups within the ROS, which is consistent with our findings for GelMA, as both are biomolecule‐based hydrogels. After washing the samples with MilliQ water and air‐drying, peel‐off tests revealed an adhesion strength of 193.03 ± 19.59 N m^−1^ (Figure 8g‐ii). Since chitosan still remained on the APPJ‐treated LDPE after peel‐off tests, the actual adhesion between chitosan and the surfaces is higher than the measured adhesion (Movie S12, Supporting Information). ATR‐FTIR analysis confirmed the presence of chitosan on the APPJ‐treated LDPE surfaces after peel‐off tests (Figure S10e, Supporting Information). To transform the firmly attached dried chitosan monomer layer into a hydrated hydrogel, samples were immersed in NaOH, serving as an ionic crosslinker. Incubation in basic solutions, for up to 3 h, crosslinked the chitosan as they did not dissolve in PBS (Figure 8g‐iii; Movie S13, Supporting Information), demonstrating the versatility and effectiveness of our method in attaching chemically crosslinkable hydrogels to solid polymeric surfaces. Further research is needed to explore the potential role of the EIEC strategy to coat hydrogel layers on metallic surfaces coated with a dense layer of carbon using ion‐assisted plasma polymerization techniques.

Overall, the fabrication of HSH constructs using the APPJ‐EIEC strategy opens up the possibility of creating various hydrogel coatings, such as photo‐crosslinkable or chemically crosslinkable hydrogels, on different solid polymeric materials in a single step without the need for chemical linkers. While this study focused on polymeric substrates with carbon‐based backbones, the approach could be expanded to inorganic substrates such as metals and ceramics by first depositing a polymeric interfacial layer, which can then be activated by APPJ to enable the fabrication of HSH constructs. The HSH constructs can be biofunctionalized by incorporating specific bioactive reagents, such as growth factors and antimicrobial agents, during the rehydration phase, making them suitable for specific tissue regeneration and personalized medicine applications. The ultimate applications of these hybrid materials could range from bone implants and wound dressings to biosensors, 3D cell‐culturing platforms, food packaging, and beyond.

## 3 Conclusion

Many methods have been recently developed to anchor hydrogels to solid materials, yet these approaches often involve complex, multi‐step processes and toxic chemicals, raising concerns about scalability, reproducibility, and environmental impact. There is a need for simpler, eco‐friendly methods to create robust hybrid solid‐hydrogel (HSH) constructs. Here, we provided a universal, single‐step, reagent‐free method for covalent attachment of hydrogels to polymeric surfaces, including low‐density polyethylene (LDPE), polycaprolactone (PCL), and polytetrafluoroethylene (PTFE). Utilizing an atmospheric pressure plasma jet (APPJ) as a dry, versatile surface treatment, we generated reactive oxygen species (ROS) on various polymeric surfaces, providing anchorage sites for covalent hydrogel attachment. Evidence was provided that this strategy is substrate‐independent and works on diverse polymers with different chemical compositions. Hydrogels such as gelatin methacryloyl (GelMA) and chitosan were covalently bonded to the functionalized surfaces, showing durability even after strong detergent washing. It was demonstrated that adjusting the hydrogel solution pH and solution ionic strength can enhance covalent bonding by increasing monomer arrival rate to the ROS‐functionalized surfaces. The effectiveness of the linker‐free covalent bonding was further amplified by an evaporation‐induced enhanced concentration (EIEC) strategy. This approach increased the likelihood of reactions between hydrogel monomers and ROS, resulting in robust hydrogel coatings with adhesion strength up to 60 kPa in wet conditions, significantly surpassing that achieved through previously reported silane‐based surface modifications. The hybrid materials demonstrated significant stability in aqueous media, could be stored dehydrated and rehydrated as needed, while maintaining structural integrity and adhesion strength. Cell experiments confirmed the cytocompatibility of the hybrid materials, with no adverse reactions observed. This approach offers a fast, polymer‐independent method for creating macro/millimeter‐thick HSH structures with enhanced covalent bonds, without the need for extra coupling agents, simplifying the process and improving regulatory approval prospects.

## 4 Experimental Section

Details of materials and experimental procedures are provided in Supporting Information.

## Conflict of Interest

The Authors declare no conflict of interest.

## Supporting information



Supporting Information

Supplemental Movie 1

Supplemental Movie 2

Supplemental Movie 3

Supplemental Movie 4

Supplemental Movie 5

Supplemental Movie 6

Supplemental Movie 7

Supplemental Movie 8

Supplemental Movie 9

Supplemental Movie 10

Supplemental Movie 11

Supplemental Movie 12

Supplemental Movie 13

## Data Availability

The data that support the findings of this study are available from the corresponding author upon reasonable request.
