# Supplementary material for: Universal Method for Covalent Attachment of Hydrogels to Diverse Polymeric Surfaces for Biomedical Applications
Source: Adv Mater. 2025 Aug 7;38(1):e03524. doi: 10.1002/adma.202503524 (PMC12759228; doi:10.1002/adma.202503524)
Supplement: Supplementary file 1 — Supporting Information [file ADMA-38-e03524-s009.docx]

**Supporting Information**

**Universal Method for Covalent Attachment of Hydrogels to Diverse Polymeric Surfaces for Biomedical Applications**

Masoud Zhianmanesh^1^, Azin Khodaei^2^, Matthew Crago^3^, Oliver Lotz^1^, Sina Naficy^3,4^, Fariba Dehghani^3,4^, Marcela M.M. Bilek^1,4,5,6^, Saber Amin Yavari^2,7^, and Behnam Akhavan*^1,4,5,8,9^

*^1^. School of Biomedical Engineering, The University of Sydney, Sydney, NSW 2006, Australia.*

*^2^. Department of Orthopedics, University Medical Centre Utrecht, 3584 Utrecht, The Netherlands.*

*^3^. School of Chemical and Biomolecular Engineering, The University of Sydney, Sydney, NSW 2006, Australia.*

*^4^. The University of Sydney Nano Institute, University of Sydney, Sydney, NSW 2006, Australia.*

*^5^. School of Physics, University of Sydney, Sydney, NSW 2006, Australia.*

*^6^. Charles Perkins Centre, University of Sydney, Sydney, NSW 2006, Australia.*

*^7^. Regenerative Medicine Center Utrecht, University Medical Center Utrecht, 3584 Utrecht, The Netherlands.*

*^8^. School of Engineering, University of Newcastle, Callaghan, NSW 2308, Australia.*

*^9^. Hunter Medical Research Institute (HMRI), New Lambton Heights, NSW 2305, Australia.*

** Corresponding Author:* [*Behnam.Akhavan@Newcastle.edu.au*](mailto:Behnam.Akhavan@Newcastle.edu.au)*;* [*behnam.akhavan@sydney.edu.au*](mailto:behnam.akhavan@sydney.edu.au)

# Materials and Experimental Sections

## Materials

Low-density polyethylene (LDPE) and polytetrafluoroethylene (PTFE), both with a thickness of 0.2 mm, were purchased from Good Fellow, UK. Type A gelatin from porcine skin (300 bloom), methacrylic anhydride (94%), 2-hydroxy-1-(4-(2-hydroxyethoxy) phenyl)-2-methyl-1-propanone (Irgacure 2959, 98%), rhodamine B for fluorescence, chitosan (medium molecular weight), sodium hydroxide (NaOH 98%), acetic acid (99%), 3-(Trimethoxysilyl)propyl methacrylate (TMSPMA), Gelatin methacryloyl (GelMA) with 80% and 40% degree of methacrylation, 2,2,3,3,4,4,4-Heptafluorobutylamine (96%), N-(3-Dimethylaminopropyl)-N'-ethylcarbodiimide (EDC), N-Hydroxysuccinimide (NHS), and phosphate-buffered saline (PBS) tablets were all purchased from Sigma Aldrich. Sodium dodecyl sulphate (SDS) was purchased from Merck KGaA, 64271 Darmstadt. Facilan ^TM^ polycaprolactone (PCL) 100 was purchased from 3D4makers B.V. to fabricate PCL thin films. High-purity helium gas was obtained from BOC Australia.

## Atmospheric pressure plasma jet (APPJ) surface functionalisation

A custom-made APPJ system, schematically depicted in Figure S1a, was employed to generate reactive oxygen species (ROS) on the polymeric surfaces. Plasma was generated within a glass tube wrapped with two separate copper electrodes, each measuring 1.5 cm in length. The ground electrode was positioned upstream, while the active electrode was located downstream, with a separation of 2 cm between them. The distance from the active electrode to the bottom of the plasma nozzle was 5 mm. Helium was selected as the plasma gas due to the lower breakdown voltage compared to other gases like argon and nitrogen [1, 2]. Plasma parameters were selected as described previously [3]. Briefly, the gas flow rate was set to 1.9 L/min, allowing a uniform plasma plume. To induce breakdown, a high AC voltage with an amplitude of 4.5-4.6 kV was applied to the powered electrode. The resonance frequency was adjusted to 32.5 kHz. The APPJ was mounted on a 3D printer (F1Sun i3 Prusa) modified in-house, allowing movement in three XYZ directions. Repetier software was used to control the movement of the plasma nozzle. The imported G-code enabled scanning of consistent parallel lines with a 1 mm spacing, allowing plasma to functionalise the entire surface. All the polymeric surfaces in this study were APPJ-treated with a constant plasma nozzle-to-surface distance of 10 mm. For the covalent immobilisation of gelatin-rhodamine B, LDPE sheets were subjected to a single-point APPJ treatment at a nozzle-to-surface distance of 10 mm for 15 seconds using the same gas flow rate and voltage parameters as above.

## GelMA synthesis procedure

GelMA was synthesised following previous methods [4]. Briefly, gelatin type A sourced from porcine skin was solubilised in PBS (pH 7.4) at a concentration of 10% (w/v). The solubilisation process involved magnetic stirring at 50 °C for 1 hour until a clear solution was obtained. Methacrylic anhydride (MA) was then added drop by drop to the gelatin solution, gradually reaching the desired volume at a rate of 0.5 ml/min. The addition of MA was carried out under rigorous stirred conditions (500 rpm), and the total solution was maintained at 50 °C for 2 hours. To minimise the influence of room light, the solution container was covered with aluminium foil. Finally, the reaction was halted by diluting the mixture fivefold with PBS heated to 50 °C. The mixture was dialysed using a molecular weight cutoff of 12-14 kDa dialysis tubes at a temperature of 40 °C to prevent polymer gelation inside the dialysis tubes. After 7 days of dialysis, the polymer solution was stored in the -80 °C freezer for two days and then freeze-dried using a Martin Christ Freeze Dryer.

## Conjugation of rhodamine B on gelatin

Rhodamine B was conjugated to gelatin using EDC/NHS chemistry through a standard catalytic reaction. Initially, 10 mL of 1% (w/v) Rb was prepared by dissolving 100 mg Rb in MilliQ water. Subsequently, 32 mg of EDC and 24 mg of NHS were added to the rhodamine B-containing solution to activate the carboxyl functional groups of rhodamine B. The final solution was stirred overnight, with the pH adjusted to 5.5. Gelatin at 2% (w/v) was prepared by dissolving 2g gelatin in 100 mL MilliQ water. A volume of 3.2 mL of rhodamine B solution was added to the gelatin solution. Stirring was maintained for an additional 24 hours, with the pH adjusted to 8.5. On the final day, the pH was adjusted to ~7 using 0.1M HCl. The resulting product was then transferred to a dialysis bag (MW cut off: 12–14 kDa, Fisher Scientific). Dialysis was performed for 7 days to remove unreacted EDC/NHS and unconjugated rhodamine B, followed by filtration through a 0.2 µm filter to obtain a clear gelatin-rhodamine B solution.

## Covalent immobilisation of biomolecules, including GelMA, gelatin, chitosan, gelatin-rhodamine B, and fluorinated probe molecules on LDPE substrates

To evaluate the covalent attachment of biomolecules on LDPE substrates, an incubation and washing protocols were used as follows. LDPE surfaces were cut in dimensions of 1 × 2.5 cm (and 15 mm × 30 mm only for gelatin-Rb attachment) and placed in Polydimethylsiloxane (PDMS) molds (1 mm thickness) for the incubation process. Unless otherwise specified, all biomolecule solutions were prepared at 2% (w/v) concentration in appropriate solvents and volumes to cover all the surfaces and applied to the LDPE substrates immediately after APPJ treatment. Samples were wrapped in aluminium foil and left overnight in a 37 °C incubator, followed by washing with MilliQ water at 37 °C for 30 minutes. To assess the nature of covalent bonding, selected samples were washed with 2% (w/v) SDS detergent solution at 65 °C for 30 minutes. SDS is an ionic detergent, known for its ability to disrupt physical interactions, including electrostatic forces, hydrogen bonds, and Van der Waals forces, while leaving covalent bonds unaffected [5, 6]. After SDS washing, the washed samples were again rinsed thoroughly with Milli-Q water. A nitrogen gas flow was used to dry the samples before surface characterisation. The following molecules and conditions were tested:

*GelMA (degree of methacrylation: 66.5%):* 2% (w/v) GelMA was dissolved in PBS (pH ~7) and immobilised on both untreated and APPJ-treated LDPE and PCL surfaces (PCL substrates were prepared identically to the procedure used for LDPE). A total of 6 samples per group were prepared for the immobilisation of GelMA on LDPE surfaces, and a total of 3 samples per group were used for the immobilisation of GelMA on PCL surfaces.

*GelMA with degrees of methacrylation of 40% and 80%:* This experiment was conducted to assess the effect of the degree of methacrylation (DoM) on covalent immobilisation. GelMA variants with DoM values of 40% and 80% were dissolved in PBS and immobilised following the same procedure. Each condition included 4 replicates.

*GelMA under different ionic strengths:* PBS was prepared by dissolving two tablets in 200 mL Milli-Q water (yielding ~300 mM ionic strength) and then diluted to ~15 mM. This buffer was used to dissolve 2% (w/v) GelMA for evaluating the impact of ionic strength on immobilisation. Each condition included 3 replicates.

*Gelatin and chitosan:* To investigate the effect of charge-charge interactions on covalent attachment, gelatin was dissolved in PBS (pH ~ 7) and in 0.5 M NaOH (pH ~ 12), while chitosan was dissolved in 2% acetic acid (pH ~ 3). Each condition included 3 replicates.

*Gelatin-Rhodamine B (Gelatin-Rb):* To enable fluorescence-based validation of covalent bonding, 2% (w/v) gelatin-rhodamine B was prepared and immobilised on APPJ-treated LDPE surfaces following the same overnight incubation and SDS washing procedure. Each condition included 3 replicates.

*2,2,3,3,4,4,4-heptafluorobutylamine (fluorinated probe molecule with a primary amine group):* A 2% (v/v) solution of this primary amine-functionalised molecule was applied to APPJ-treated LDPE to occupy and block the reactive oxygen-containing functional groups (OFGs). After overnight incubation and SDS washing, a set of samples was subsequently coated with 2% (w/v) GelMA and subjected to the same immobilisation and washing steps. Each condition included 4 replicates.

## X-ray Photoelectron Spectroscopy (XPS) measurements

XPS measurements for evaluating the surface chemistry of samples were carried out using a Thermo Scientific TM K-Alpha+TM spectrometer (Thermo Fisher Scientific, UK), and data collection and analysis were performed using Thermo Avantage software (version 5.9902, Thermo Fisher Scientific, UK). The X-ray source employed was a monochromatic Al K-Alpha (hν =1486.6 eV) operating at a nominal voltage of 12.0 eV. The electron take-off angle was set to 90°, referenced to the samples, and measurements were conducted at base pressure below 5.0 × 10^-8^ Pa. The dried samples were mounted on the XPS sample holder using conductive carbon tape. Survey spectra were obtained and recorded in an energy range of 9.92 eV to 1350.08 eV with a step size of 1.0 eV. The step size was set to 0.1 eV for high-resolution carbon (C1s) with a total of fifteen scans collected in an energy range of 280 eV to 300 eV. The spot size was set to 400 µm and 40 µm once the charging effect was observed. The spectra were charge-corrected in accordance with the binding energy (BE) of aliphatic carbon (284.6 eV). All XPS measurements were performed approximately 24 hours after the immobilisation process and were conducted at three randomly selected points on each sample.

## Attenuated Total Reflection-Fourier-Transform Infrared (ATR-FTIR) Spectroscopy measurements

ATR-FTIR measurements were carried out using a Bruker Lumos stand-alone FTIR microscope equipped with a liquid nitrogen-cooled MCT detector. The infrared analysis was performed using a germanium ATR crystal with a high refractive index. The ATR crystal was controlled by precise piezo-electrical motors integrated into the lens, allowing accurate positioning of the ATR crystal on the samples. The applied contact pressure of the crystal was set to “medium pressure.” To obtain a sufficient signal-to-noise ratio, each spectrum was compiled from 256 scans with a resolution of 4.0 cm^−1^ in the wavenumber range of 4000–600 cm^−1^. The data obtained were processed using the instrument’s OPUS v8.2.21 software. To compare the results between the experimental conditions, all spectra were normalised based on the intensity of their corresponding highest peak prior to subtraction. To ensure consistency, the measurements were taken from three random points on each sample.

## Zeta potential measurements

Zeta potential measurements were conducted using a SurPASS system (Anton Paar, Germany) at ambient temperature. Samples sized 15 mm × 30 mm were initially treated and subsequently trimmed to 12 mm × 24 mm to fit within the flow cell. The electrolyte employed was a KCl solution (~1 × 10^-3^ M), and the streaming potential was adjusted using 0.05 M HCl across a pressure range of 400 mbar. Appropriate volumes of HCl were added to the electrolyte solution, reducing the pH by ~ 0.5 unit steps. Four measurements were taken for each pH value, with a minimum of two sample sets assessed for each condition.

## Contact angle measurements and surface free energy (SFE) calculations

Contact angle measurements were carried out using the sessile drop method with a contact angle goniometer (Attension Theta, Biolin Scientific). A droplet of 0.3 ml of deionised water (as a polar liquid) and/or diiodomethane (as a nonpolar liquid) was placed on the samples. Three repeats with different positions per sample were selected, and average values with corresponding standard deviations were reported. Side-on images were captured to determine the contact angles for each sample. The SFE and its polar and dispersive components were calculated using the Owens-Wendt-Rabel-Kaelble model and Attension Theta software.

## Fluorescence microscopy

The immobilised gelatin-rhodamine B on untreated and APPJ-treated LDPE surfaces was visualised using a Nikon Ni-E Basic Widefield microscope in fluorescent mode. Multiple images were captured using a Fluor 4X lens from each sample and merged using NIS-Elements AR software, with post-processing performed in ImageJ. The quantified fluorescence signals obtained from Image J were then normalised based on the APPJ-treated before SDS washing.

## Fabrication of micro/millimetre-thick layer of GelMa and Chitosan hydrogels on polymeric surfaces

Various polymeric surfaces (LDPE, PCL, and PTFE) were APPJ-treated using the same procedure and plasma parameters as described in section 2.2. Appropriate volumes of GelMA solution with concentrations of 10% (w/v) and 5% (w/v), were prepared by dissolving GelMA in PBS containing 0.5% (w/v) Irgacure. The hydrogels were incubated on the polymer surfaces and crosslinked using UV light (365 nm), followed by a 48-hour drying process in an incubator set at 37°C. Samples were then rehydrated in PBS to form a hydrated gel layer on the polymeric surfaces.

To form a micro/millimetre-thick layer of hydrated chitosan hydrogel on LDPE surfaces, 2% (w/v) chitosan was dissolved in a 1% acetic acid solution with a pH of 3. Subsequently, the pH of the solution was adjusted to ~7 using NaOH. Then, 300 µL of chitosan solution was added to the APPJ-treated LDPE (5 cm × 1 cm) for covalent immobilisation. The samples were dried in an incubator at 37°C for 48 hours and washed multiple times with water to remove unbound chitosan monomers. Following another drying phase, the samples were incubated in 30% (w/v) NaOH for crosslinking while covalently bonded to the APPJ-treated LDPE.

## Aqueous stability tests: Static and Dynamic conditions

Water stability tests were conducted using both static and dynamic methods. In the static test, 5% (w/v) GelMA was dissolved in PBS containing 0.5% Irgacure. GelMA solution with a volume of 150 µL was added to each of the untreated and APPJ-treated LDPE (3 samples per condition, each measuring 1.5 cm × 1 cm). The samples were exposed to UV light at 365 nm for 20 minutes. The samples were then immediately immersed in 10 ml PBS (pH ~ 7) for specified durations (3 days, 1 week, 2 weeks, 1 month and 2 months) and in cell culture medium (RPMI 1640) for 1 day, 1 week, and 1 month. After each time point, the corresponding samples were removed, dried, and stored for subsequent characterisation.

For the dynamic test, a custom-made fluidic device, schematically shown in Figure S1b, was used to evaluate the retention and stability of the GelMA hydrogel on untreated and APPJ-treated LDPE. GelMA hydrogels were formed on untreated and APPJ-treated LDPE using the same protocol and sample numbers as for the static test. For this test, a GelMA solution with a volume of 80 µL was placed on each LDPE. The samples coated with GelMA hydrogels were then placed face down on a PDMS sheet, which has a rectangular cutout in the middle to create a channel for the flow of PBS beneath the surface. A Masterflex L/S pump was employed to circulate PBS in the system. The pump was operated at 107 rpm, resulting in a flow rate of 50 ml per minute for 12 hours within a polystyrene tube with an inner diameter of 3.5 mm. Following completion, the samples were dried for subsequent characterisation.

## Swelling ratio measurements

The swelling kinetics of GelMA hydrogel coated on LDPE surfaces were evaluated to investigate the swelling of air-dried hydrogels. Briefly, 150 µL of GelMA hydrogel, containing 10% (w/v) GelMA and Irgacure 0.5% (w/v), was formed on three untreated and three APPJ-treated LDPE surfaces (LDPE were cut with dimensions of 1.5 cm ×1 cm) using UV light at 365 nm for 20 minutes to induce gelation. The samples were then weighed and named as *W_w_*. Subsequently, the samples were placed in an incubator at 37°C for 48 hours to ensure that the hydrogel coatings were thoroughly dried. The samples were weighed multiple times until constant values were reached, and the final dried weight was recorded as *W_d_*. The dried HSH constructs were then incubated in 8 mL of PBS (pH ~ 7) solutions within 6-well plates. The weights of swollen hydrogels were recorded at various time points (up to a week), and the corresponding swelling ratio was calculated using Equation 1. Additionally, water retention was calculated to compare the final weight of samples in their wet state with their original weight before drying (Equation 2).

Swelling ratio = $\frac{W_{t}- W_{d}}{W_{d}} \times100$ Equation 1

Retention capacity = $\frac{W_{t}}{W_{w}} \times100$ Equation 2

Where *W_t_* is the weight of rehydrated samples after each time point, *W_d_* is the weight of the dried sample at the initial point, and *W_w_* is the weight of the wet samples at the initial point.

## Indentation testing of as-made and rehydrated GelMA hydrogel

The mechanical properties of GelMA hydrogels before and after a drying/rehydration (D/R) cycle were assessed using indentation testing. GelMA hydrogels 10% (w/v) were prepared in PBS containing 0.5% (w/v) Irgacure 2959, added into APPJ-treated LDPE samples (15 mm × 10 mm), and crosslinked under UV light. Two groups of samples were prepared (n = 5 per group): (i) as-made hydrogels, incubated in PBS at 37 °C for 1 hour before testing, and (ii) rehydrated hydrogels that had been previously dried at 37 °C for 48 hours and subsequently rehydrated in PBS overnight. On the test day, all samples were gently blotted using Kimwipes to remove surface moisture and left briefly at room temperature to allow slight surface drying prior to testing.

Indentation measurements were performed using a uniaxial mechanical testing system (Instron BioPuls 5943, USA) equipped with a stainless-steel hemispherical tip of 4 mm diameter. The probe was initially brought into contact with the hydrogel surface until a preload of 0.01 N was reached, determining the zero-indentation baseline. The probe was then further displaced into the hydrogel to a fixed depth of 0.4 mm, and the corresponding reaction force was recorded as an indicator of the hydrogel’s stiffness and resistance to deformation.

## Adhesion strength measurements

### Peel-off test

Peel-off mechanical tests, schematically depicted in Figure S1c, were conducted to investigate the adhesion strength of the GelMA hydrogel coated on untreated and APPJ-treated polymeric surfaces. Briefly, 10% (w/v) GelMA was dissolved in the PBS solution containing 0.5% (w/v) Irgacure as the initiator. LDPE substrates were cut with dimensions of 4 cm × 1 cm and were APPJ-treated. 200 µL of GelMA solution was pipetted on the substrates, followed by UV light (365 nm) irradiation for 20 minutes to form GelMA hydrogels. Using this procedure, 4 untreated and 8 APPJ-treated LDPE samples were prepared. The samples were then stored at 37°C for 48 hours for drying. 4 samples from APPJ-treated groups were deliberately scratched using a razor blade to further investigate the effect of physical stress and surface damage on adhesion and the consistency of coatings. The same protocol was used to test the GelMA adhesion coated on PCL and PTFE substrates.

Chitosan hydrogel was formed on LDPE surfaces using the same protocol as GelMA hydrogel. Briefly, 2% (w/v) chitosan was dissolved in 1% acetic acid with a pH of 3. The pH of the solution was adjusted to ~7 by dropwise addition of NaOH (5% w/v) solution. Then, a volume of 200 µL of chitosan solution was added to untreated and APPJ-treated LDPE surfaces (4 samples per group with a dimension of 4 cm × 1 cm). These samples were dried and then washed multiple times with MilliQ water to remove any unbound chitosan monomers. Following another drying cycle, the samples were used for peel-off tests.

The peel-off tests were performed using a standard polyester/non-woven tape strip (Intertape Polymer Group, Florida, US). A uniaxial mechanical testing machine (Instron BioPuls 5943, US) was employed to peel the tape strips from the samples at a constant rate of 10 mm per minute. The tape strips were placed precisely on the dried hydrogels without any interaction with the bottom surfaces. The tape strip was then connected to the upper clamp of the uniaxial mechanical tester and pulled away perpendicular to the substrate. All samples were firmly placed on a glass plate using double-sided tape and super glue, and the glass slides were firmly attached to the lower clamp of the mechanical tester. Force per unit width was calculated to allow comparison between different experimental groups.

### Single-LAP shear test

Single-LAP shear tests were conducted to examine the adhesion strength between GelMA hydrogel and LDPE surfaces in hydrated and dehydrated forms (Figure S1d). Untreated and APPJ-treated LDPE surfaces were cut into dimensions of 1 cm × 5 cm. GelMA solution with a concentration of 10% (w/v) containing 0.5% (w/v) Irgacure was prepared in PBS. LDPE surfaces were positioned on a glass plate, and a rectangular silicone mold (2 cm × 1 cm) with a thickness of 1 mm was placed on the substrates. Subsequently, 200 µL of GelMA solution was pipetted within the mold on top of the first LDPE surface (with the APPJ-treated side facing up). Another LDPE surface (with the APPJ-treated side facing down) was immediately placed on top, enclosing the GelMA solution. The samples were gently placed inside a UV box and exposed to UV light at 365 nm for 30 minutes for gelation. At the halfway point, the samples were flipped inside the UV treatment box to ensure uniform crosslinking. This setup resulted in the GelMA hydrogel, with a thickness of ~ 1 mm, being sandwiched between two LDPE substrates.

For hydrated forms, untreated LDPE with GelMA and one set of APPJ-treated LDPE with GelMA were immersed in PBS immediately after being removed from the UV box for 3 hours prior to conducting the LAP shear tests. This precaution was taken to minimise the dehydration of the hydrogel at room temperature and to remove non-crosslinked polymer chains. One set of samples containing GelMA sandwiched between two APPJ-treated LDPE sheets was dried for 48 hours at 37°C and rehydrated in PBS for three days before testing. Five samples per group were created for statistical analysis. To compare the adhesion strength of GelMA hydrogel directly attached to ROS-functionalised surfaces without chemical linkers, a comparison was made with a control set of samples utilising linker molecules. Following the protocol suggested by Yuk *et al*. [7], a silanisation process with TMSPMA was conducted to form GelMa hydrogels layer on ROS-functionalised LDPE surfaces. Briefly, APPJ-treated LDPE surfaces were immediately incubated in 5 ml silane solution (100 ml deionised water, 10 µl of acetic acid with pH 3.5 and 2 wt% of TMSPMA) for 2 hours at room temperature after APPJ treatment. Samples were removed, washed with ethanol, and dried. The GelMA 10% (w/v) solution containing 0.5% (w/v) Irgacure, with a volume of 200 µL, was sandwiched between two silane-functionalised LDPE surfaces and crosslinked using UV light at 365 nm for 30 minutes. Five samples were immersed in PBS right after being removed from the UV box, and five samples were dried for 48 hours at 37°C and rehydrated in PBS for three days before testing.

For dehydrated forms, a set of four samples involving GelMA hydrogels sandwiched between two APPJ-treated LDPE surfaces was dried for 48 hours in an incubator at 37°C before conducting the LAP shear tests.

A uniaxial mechanical testing machine (Instron BioPuls 5943, US) was employed to apply force to one LDPE piece by pulling it parallel to the plane of adhesion between the two surfaces at a rate of 10 mm per minute, while keeping the other piece fixed in place. To convert the force-displacement curves to stress-strain curves, the force values were divided by the surface area covered by hydrogels (F/A), and the displacement values were divided by the length of the covered area (mm/mm).

### Scrape test

A custom-made mechanical test, referred to here as the “Scrape Test”, was designed to compare the adhesion strength of coatings between samples prepared under different conditions (Figure S1e). A 10% (w/v) GelMA hydrogel containing 0.5% (w/v) Irgacure with a volume of 150 µL was added to both APPJ-treated and untreated LDPE surfaces. The samples were then placed in an incubator at 37 °C for 3 hours before being exposed to UV light. Thereafter, gelation occurred by exposure to 365 nm UV light for 20 minutes. Another set of samples was prepared by subjecting the APPJ-treated LDPE samples coated with GelMA to a drying process. Three samples were also scratched with a razor blade. The thickness of GelMA formed on the sample was measured to be ~3 mm after being removed from the UV box and ~ 1 mm after drying/rehydrating cycles. All the samples were then immersed in water for 3 days before conducting the Scrape tests.

To perform the Scrape Test, each sample was individually attached to a piece of a microscope glass slide from the LDPE side using double-sided tape. A 3D-printed setup (Figure S1e) was fabricated to pull the hydrogel up from the LDPE surfaces. Grease was applied to the edge of the glass slide to minimise friction between the glass and the 3D-printed device. A vertical force was applied to the 3D-printed device to pull the GelMA hydrogel up from each sample, using a uniaxial mechanical testing machine (Instron BioPuls 5943, US) at a rate of 10 mm/min. Adhesion strength was determined from force-displacement data and the area covered by the GelMA hydrogel. A total of four samples were prepared for each condition.

## *In vitro* biocompatibility

To assess the cytocompatibility of the HSH constructs, the viability of macrophages (as a phagocytic cell type) and human mesenchymal stem cells (hMSCs, as a non-phagocytic cell type) were investigated. Thin and thick layers of GelMA hydrogel were formed on APPJ-treated LDPE surfaces using 5% and 10% (w/v) GelMA containing 0.5% Irgacure with a volume of 50 µL per sample, respectively. Control samples, both untreated and APPJ-treated, were also prepared. Following UV sterilisation for 30 min, all samples were prepared for the subsequent *in vitro* study. To investigate the effect of the APPJ-EIEC strategy on hMSCs activity, the same GelMA amounts were applied to untreated and APPJ-treated LDPE samples, followed by 48 hours of drying. Samples were rehydrated in cell media (α-MEM supplemented with 10% (v/v) FBS and 1% (v/v) Pen-Strep) overnight.

THP-1, a human leukemia monocytic cell line, was initially cultured in a 75 mL flask as a cell suspension. The culture medium used was RPMI 1640 (Invitrogen, USA), containing 10% (v/v) fetal bovine serum (FBS) and 1% (v/v) Penicillin-Streptomycin (Pen-Strep) (Invitrogen, USA). Subsequently, the culture medium was supplemented with 160 nM of phorbol 12-myristate 13-acetate (PMA, ≥99% Sigma-Aldrich) for 24 h to induce differentiation into M0 phenotype macrophages. The differentiated THP-1 derived M0 macrophages were then seeded onto 48-well plates at a density of 50,000 cells per well.

hMSCs were also cultured in a 75 mL flask using α-MEM supplemented with 10% (v/v) FBS and 1% (v/v) Pen-Strep media. Subsequently, 3.000 typsinised (Invitrogen, USA) hMSCs were seeded onto each sample in a 48-well plate. After cell seeding, the cultures were maintained at a constant temperature of 37°C with a stable level of 5% CO_2_.

A tetrazolium reduction-based, WST-8/CCK cell counting kit (Abcam, UK) was applied to quantify the cell viability. The samples were incubated with 5% WST-8 solution for 2 hours, following absorbance detection at 460 nm using a multimode plate reader (Clariostar plate reader, BMG Labtech). Additionally, the DNA content of attached cells was quantified at the final time point in accordance with the manufacturer’s instructions, utilising the Quant-iT^TM^ PicoGreen kit (ThermoFisher Scientific, USA). The amount of DNA was measured after subjecting it to three cycles of freeze-thawing in lysis buffer (0.5% Triton X-100) at -80°C.

## Live-dead imaging

To verify the correlation between metabolic activity and cell viability, cellular staining was performed using a live-dead kit (Molecular Probes, ThermoScientific, US). A confocal microscope (CSLM- Leica SP8X, Germany) was employed to capture the signals emitted by live and dead cells, which were visualised in two distinct colours: green 500-525 nm) and red (528-640 nm), respectively.

## Cell adhesion

To investigate the cell adhesion behaviour and morphology of attached hMSCs to each sample, cytoskeleton staining was performed after one day of cell culture. To do this, hMSCs were cultured at a density of 20,000 cells per well on the samples within a 48-well suspension plate. After 24 h incubation, the adhered cells were fixed with formalin, and a staining protocol employing Alexa Fluor^TM^ 488 Phalloidin (Sigma-Aldrich) for actin and DAPI (Abcam, UK) for nuclei staining was utilised. Actin and nuclei were stained in red and blue, respectively, in a process referred to as cytoskeleton staining. Visualisation of the stained cells was carried out using a confocal laser microscope (CSLM- Leica SP8X, Germany).

## Immune stimulation tests

To investigate whether GelMA coatings and ROS-functionalised surfaces can stimulate immune responses, M0 phenotype macrophages, derived from THP-1 monocytes, were cultured using two distinct models. In the first model (direct method), M0 phenotype macrophages derived from THP-1 were cultured on the surface of untreated, APPJ-treated LDPE, with and without hydrogel coatings. In the second model (indirect method), macrophages were incubated in the released supernatant of the samples (1 day of incubation) in the culture medium as the condition medium. This approach aimed to discern whether the immune reaction was primarily triggered by direct contact with samples (contact mechanism) or by the materials released during degradation of the GelMA hydrogel. To assess cellular morphology, cytoskeleton staining, and subsequent visualisation were conducted on fixed cells after 1 day of incubation. Additionally, the quantification of Interleukin 6 (IL-6), an inflammatory cytokine, was performed in the conditioned medium. This was achieved using a human IL-6 ELISA kit (DuoSet, R&D Systems, USA) in accordance with the supplier’s protocol.

## Statistical analysis

All statistical analyses were performed using GraphPad Prism version 10 (GraphPad Software, LLC). Data are presented as mean ± standard error of the mean (SEM). The number of independent samples (n) for each experiment, as well as the specific statistical tests used (e.g., unpaired Student’s t-test, one-way or two-way ANOVA with Tukey’s post-hoc tests), are provided in the corresponding figure captions. Data normalisation, transformation or pre-processing (e.g., baseline correction, signal subtraction) was applied as described in the relevant experimental sections. A significance threshold of α = 0.05 was used throughout, and adjusted *P*-values are reported where comparisons were made


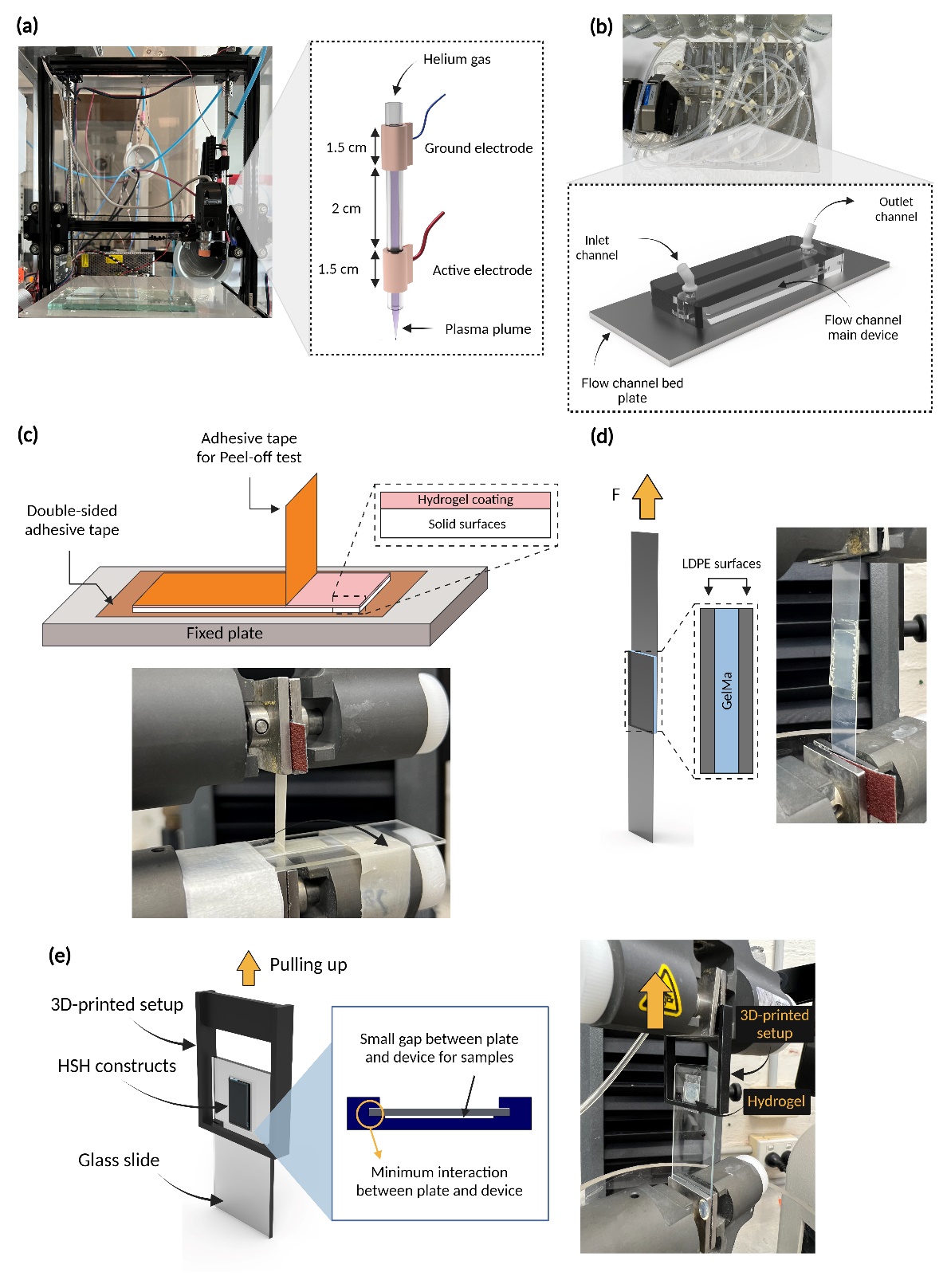


**Figure S1.** **Atmospheric pressure plasma jet (APPJ) Configuration and Custom Experimental Testing Devices. (a)** A custom-made APPJ device mounted on a 3D-printer (F1Sun i3 Prusa) modified in-house was employed for plasma surface treatment. **(b)** Schematic representation of the fluid channel employed for dynamic water stability tests. **(c)** Schematic illustration of the peel-off test procedure. Samples were fixed to a glass slide using a double-sided tape and super glue. A standard polystyrene adhesive tape was then employed to peel off dried hydrogel coatings. **(d)** Schematic representation of the single-LAP shear test. Gelatin methacryloyl (GelMA) hydrogel was sandwiched between two low-density polyethylene (LDPE) surfaces. The bottom surface was clamped, and a vertical force was applied to the other surface to induce upward movement. **(e)** A newly designed scrape test was employed to measure the adhesion strength of the GelMA coating. The surfaces with a thick layer of GelMA were placed on a glass slide using double-sided tape. A 3D-printed device was utilised to initiate motion and subsequently pull the GelMA upwards until failure occurred. To ensure that only GelMA was affected, a small gap was considered between the 3D-printed device and the glass slide, considering the thickness of the polymers and double-sided tape beneath them.

# APPJ surface treatment

Polymeric solid substrates were ROS-functionalised (reactive oxygen species-functionalised) using an atmospheric pressure plasma jet (APPJ) system for the fabrication of hybrid solid-hydrogel (HSH) constructs. Low-density polyethylene (LDPE) was selected as a model substrate due to its simple chemical composition, facilitating straightforward surface chemical characterisation after functionalisation (Figure S2a). A dynamic plasma treatment approach was employed to ensure the entire surface was uniformly treated. Treatment speeds ranged from 50 mm/min to 4000 mm/min, to determine the speed at which higher concentrations of oxygen-containing functional groups (OFGs) are achieved.

The formation of OFGs on LDPE surfaces after APPJ treatment was confirmed by X-ray photoelectron spectroscopy (XPS) and attenuated total reflectance Fourier-transform infrared (ATR-FTIR) analyses. Surface elemental compositions, calculated from XPS survey spectra (Figure S2b), showed an increase in oxygen atomic concentration (atm%) from less than 1% on untreated LDPE to 25 ± 2% on APPJ-treated surfaces at a speed of 50 mm/min (Figure S2c). Oxygen atm% decreased with increased treatment speed due to reduced exposure time to plasma. Nitrogen, at ~1.5 atm%, was detected on surfaces treated at treatment speeds below 100 mm/min. The higher incorporation of oxygen in the surface chemistry, compared to nitrogen, is due to the higher reactivity and affinity of oxygen, which dominate surface interactions and lead to the preferential formation of OFGs [8, 9]. Based on these results, the treatment speed of 200 mm/min was selected for further surface characterisation and hydrogel attachment experiments, as no significant increase in oxygen atm% was observed at lower speeds. Variations in the functional groups generated by APPJ at different treatment speeds are detailed in Figure S2d-h.


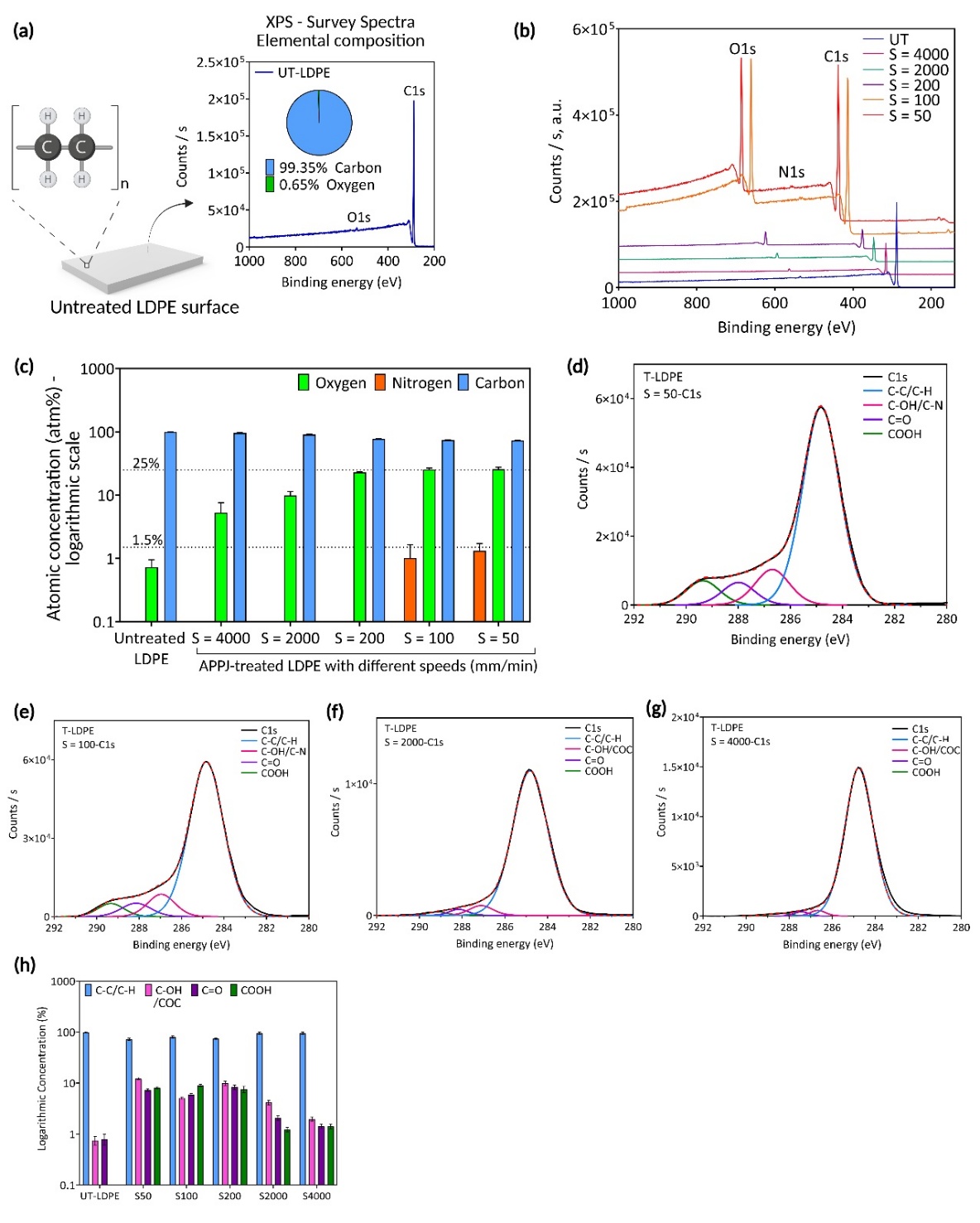


Figure S2. **Oxygen-containing functional groups (OFGs), including reactive oxygen species (ROS), are generated on the polymeric surfaces (a)** Elemental composition of untreated low-density polyethylene (LDPE) obtained from X-ray photoelectron spectroscopy (XPS) measurements. **(b)** XPS survey spectra obtained from atmospheric pressure plasma jet (APPJ)-treated LDPE surfaces at various treatment speeds. **(c)** Calculated atomic concentrations (atm%) at different plasma treatment speeds obtained from XPS survey spectra. C1s high-resolution obtained from XPS results for LDPE surface treated with **(d)** 50 mm/min, **(e)** 100 mm/min, **(f)** 2000 mm/min, and **(g)** 4000 mm/min demonstrated the presence of different OFGs at interfaces. **(h)** The concentration of OFGs created on APPJ-treated surfaces at various treatment speeds. All data presented as mean ± SEM, n=3.

ATR-FTIR results, shown in Figure S3a, are in agreement with XPS findings, showing carbonyl-containing functional groups (C=O) at 1736 cm^-1^ on both untreated and APPJ-treated LDPE. However, as observed from C1s high-resolution, the C=O presented on APPJ-treated LDPE also includes carboxyl functional groups (COOH). The broad band at 3100-3500 cm^-1^ indicated hydroxyl groups, while peaks at 2919 cm^-1^ and 2845 cm^-1^ indicated alkane groups. Subtraction of the ATR-FTIR spectrum of untreated surfaces from that of the APPJ-treated surfaces revealed the presence of C=C at 1620 cm^-1^ and C–O (ether and epoxide functional groups) at 1250 cm^-1^ (Figure S3b)

The changes in wettability as regulated by changes in surface chemistry upon APPJ treatment are shown in Figure S3c. Wettability increased with the formation of polar oxygen groups, with water contact angle (WCA) decreasing from approximately 98.9° ± 1.8 for untreated samples to 55.7° ± 2.1 for the APPJ-treated LDPE. The diiodomethane contact angle was also measured to calculate surface free energy (SFE). The results showed an increase in SFE from 29.9 ± 1.06 mN/m for untreated LDPE to 50.3 ± 1.65 mN/m for APPJ-treated LDPE.


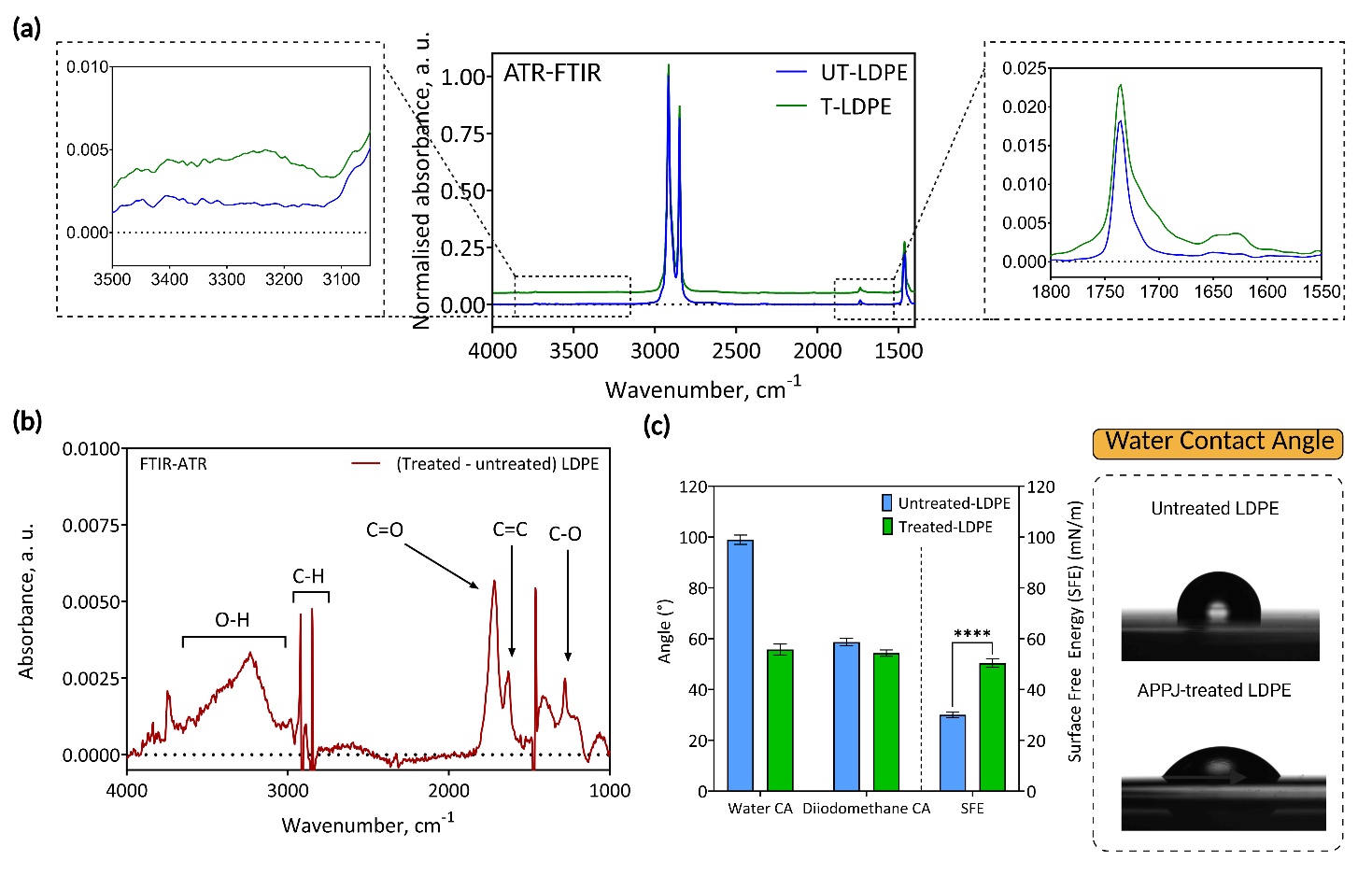


Figure S3. **Surface characterisation using attenuated total reflectance Fourier-transform infrared (ATR-FTIR) and surface free energy (SFE). (a)** The normalised ATR-FTIR results of untreated and atmospheric pressure plasma jet (APPJ)-treated surfaces showed variation in surface chemistry, as evidenced by the presence of peaks at 1640 and 1736 cm^-1^. The main strong peaks at 2915 cm^-1^ and 2848 cm^-1^ correspond to CH_2_ asymmetric stretching, and the peak at 1460 cm^-1^ is for the CH_2_ bending deformation [10]. **(b)** Upon subtraction, C=O, C–OH, C=C, and C–O functional groups were observed at 1736 cm^-1^, 3100-3500 cm^-1^, 1620 cm^-1^, and 1250 cm^-1^, respectively. **(c)** The calculated water contact angle (WCA) (left axis) and SFE (right axis) demonstrated higher SFE and lower WCA after oxygen-containing functional groups (OFGs) formation. Data presented as mean ± SEM, n=3, *P*-values are calculated using two-way ANOVA with Bonferroni correction, *****P*≤0.0001.

**Supplementary Figures**


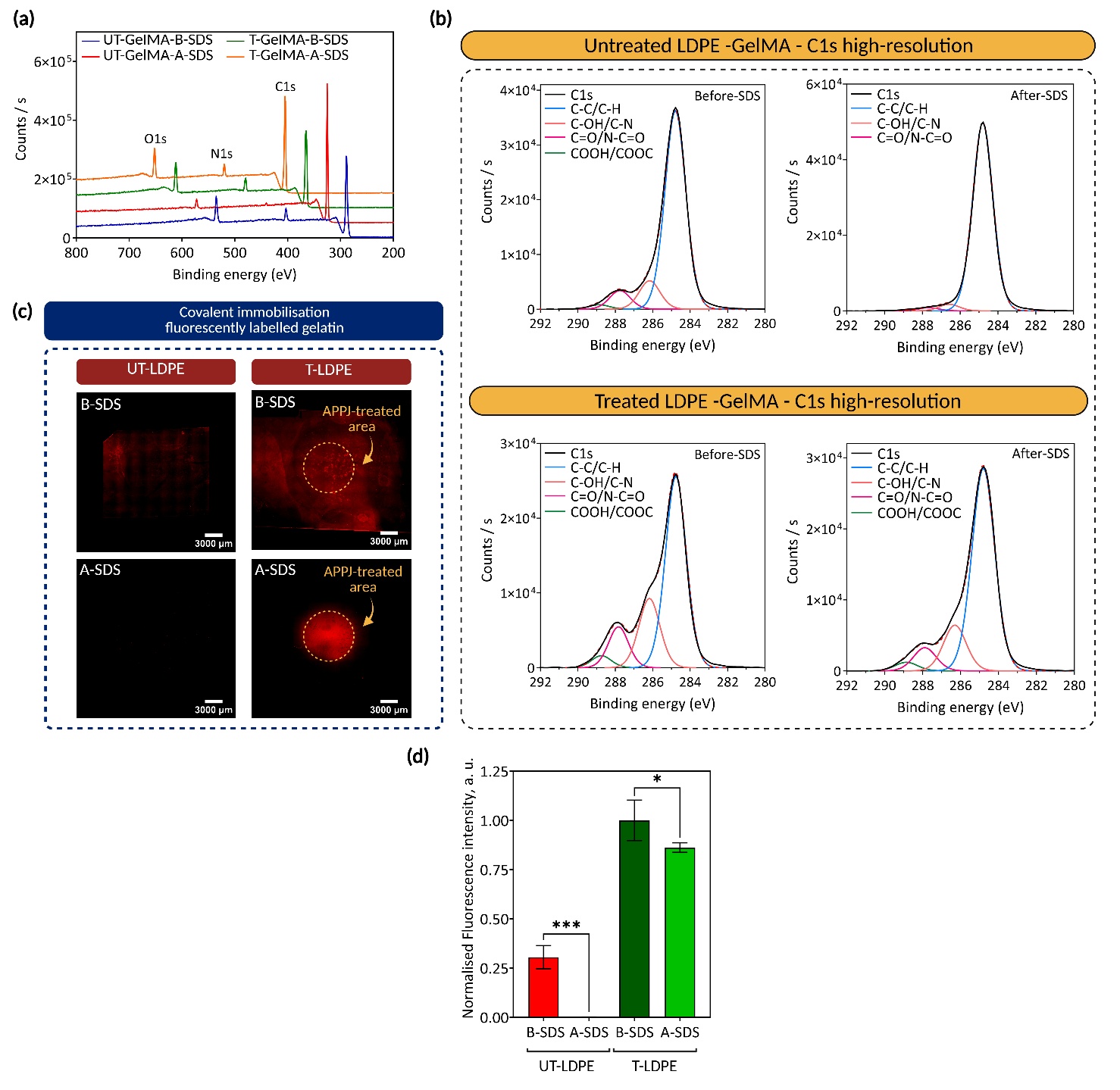


**Figure S4. Surface characterisation of gelatin methacryloyl (GelMA)-coated LDPE (low-density polyethylene) surfaces. (a)** X-ray photoelectron spectroscopy (XPS) survey spectra for the atmospheric pressure plasma jet (APPJ)-treated and untreated low-density polyethylene (LDPE) surfaces coated with GelMA before and after sodium dodecyl sulfate (SDS) washing. **(b)** C1s high-resolution spectra obtained from untreated (top) and APPJ-treated LDPE (bottom) coated with GelMA before and after SDS washing. **(c)** Fluorescence microscopy images and **(d)** normalised quantitative analysis of fluorescence intensity showing covalent immobilisation of rhodamine B-labelled gelatin on APPJ-treated (T-LDPE) LDPE surfaces after SDS washing (B-SDS: before SDS washing, A-SDS: after SDS washing). Data presented as mean ± SEM, n=3, *P*-values are calculated using two-way ANOVA using Fisher’s LSD test, ****P*≤0.001, **P*≤0.05.


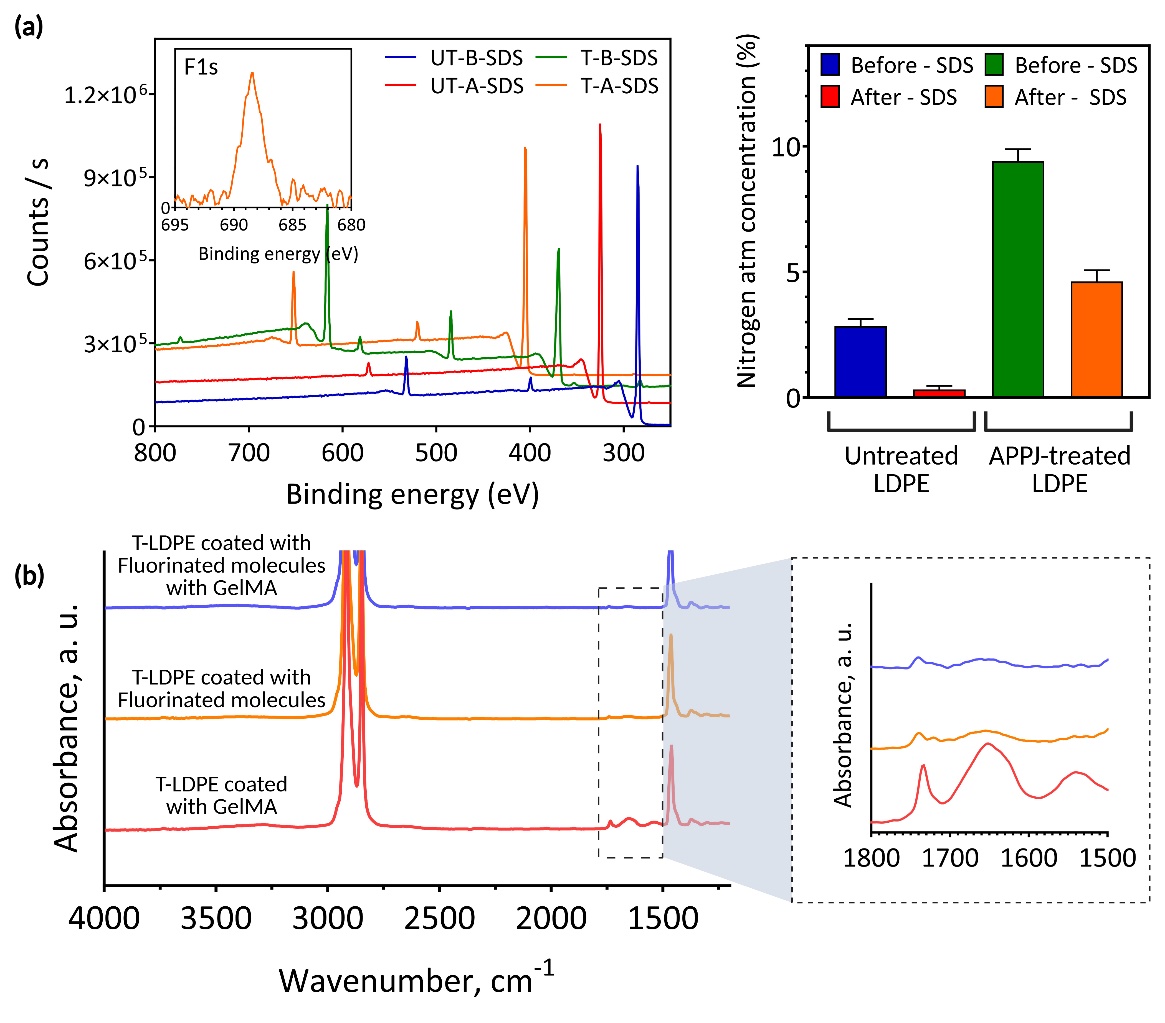


**Figure S5. Surface characterisation of low-density polyethylene (LDPE) surfaces coated with a fluorinated prob molecule with a primary amine group and gelatin methacryloyl (GelMA). (a)** X-ray photoelectron spectroscopy (XPS) survey spectra and calculated nitrogen atomic concentration (atm%) confirmed the presence of fluorinated probe molecules on atmospheric pressure plasma jet (APPJ)-treated LDPE (T-LDPE) after sodium dodecyl sulfate (SDS) washing. The detected F1s peak (inset) and nitrogen signal support successful covalent immobilisation. These fluorinated amine probes could occupy and thus block the reactive oxygen-containing functional groups (OFGs) on APPJ-treated LDPE. **(b)** Normalised attenuated total reflectance Fourier-transform infrared (ATR-FTIR) spectra show reduced amide peaks for samples coated with both fluorinated probe molecules and GelMA (blue), compared to unblocked controls (red). Data presented as mean ± SEM, n=4.


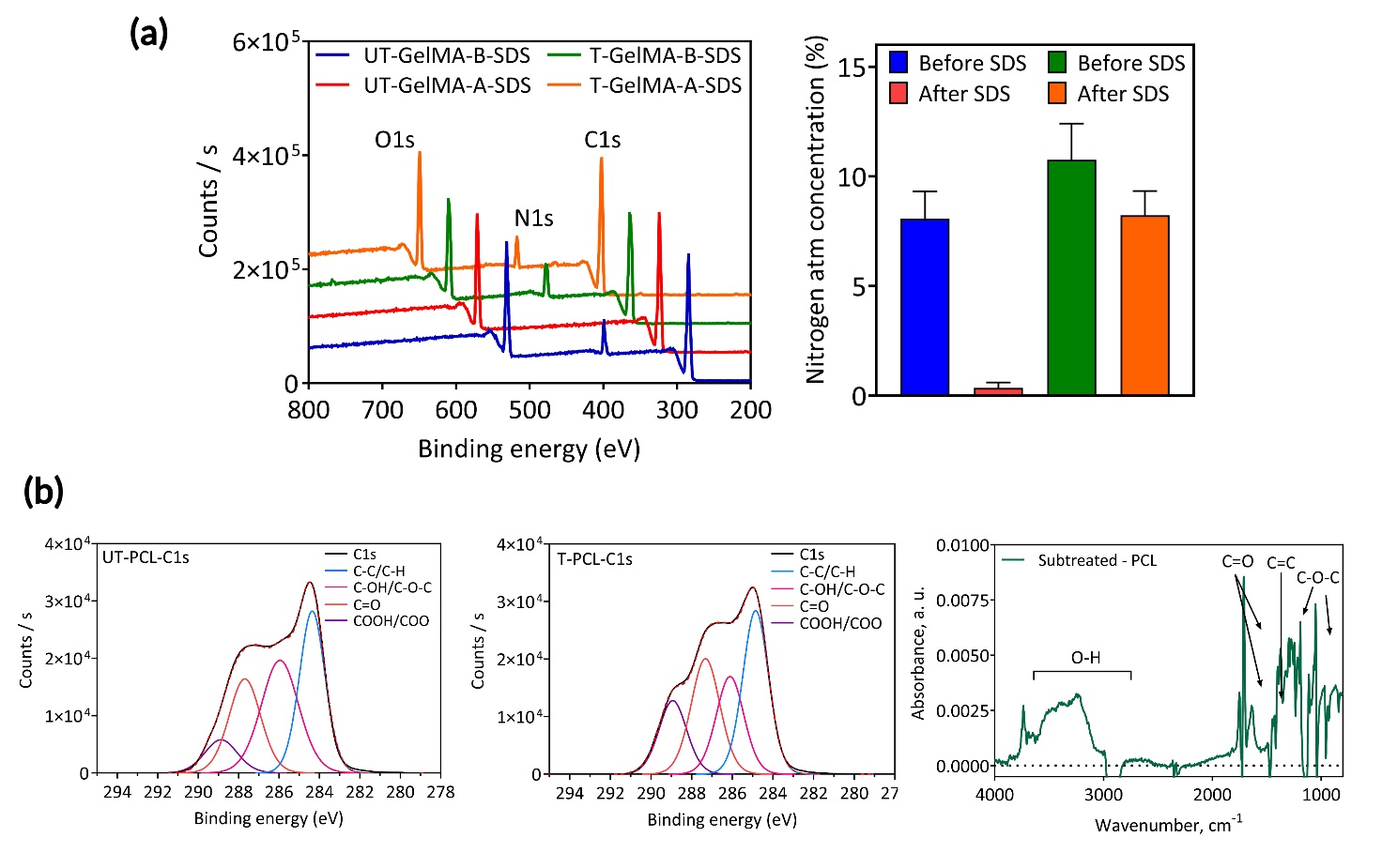


**Figure S6. Surface characterisation of untreated and atmospheric pressure plasma jet (APPJ)-treated polycaprolactone (PCL) surfaces with and without gelatin methacryloyl (GelMA) coating. (a)** X-ray photoelectron spectroscopy (XPS) survey spectra (left) and calculated nitrogen atomic concentration (atm%) for untreated (UT) and APPJ-treated (T) PCL surfaces coated with GelMA, before and after sodium dodecyl sulfate (SDS) washing. Minimal nitrogen elements were detected on untreated PCL after washing, indicating poor covalent attachment. In contrast, significant nitrogen atm% was detected on APPJ-treated PCL, supporting effective GelMA immobilisation via covalent bonding. **(b)** High-resolution XPS C1s spectra of untreated (left) and APPJ-treated (middle) PCL show the formation of various oxygen-containing functional groups (C–OH/C–O–C and COOH/COOC) after treatment. Attenuated total reflectance Fourier-transform infrared (ATR-FTIR) analysis (right panel) further confirms the presence of these oxygen-containing functional groups (OFGs), including hydroxyl, carbonyl, and ether/epoxide groups, on APPJ-treated PCL surfaces, supporting their role in facilitating covalent interactions with GelMA. Data presented as mean ± SEM, n=3.


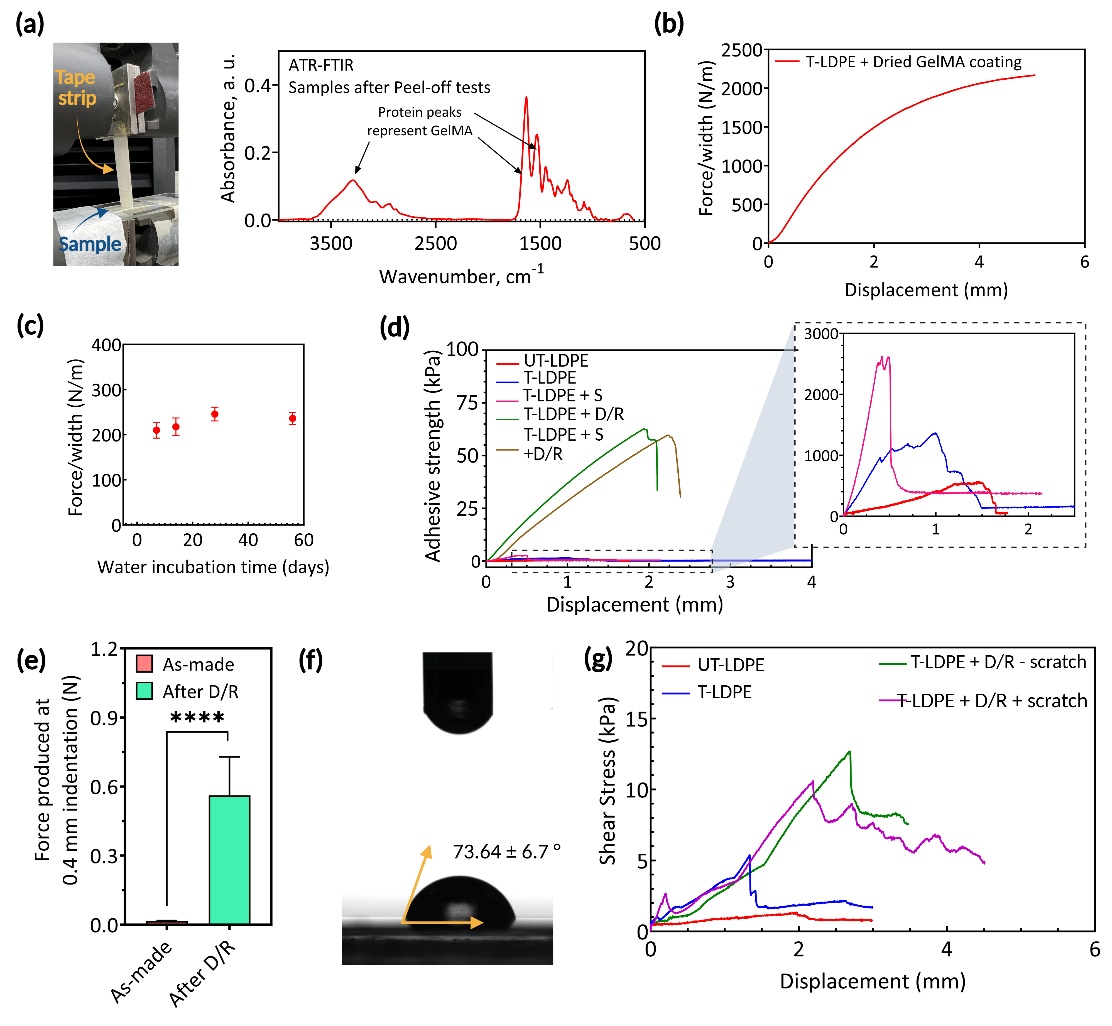


**Figure S7. (a)** Visual observation after Peel-off tests and attenuated total reflectance Fourier-transform infrared (ATR-FTIR) confirmed the presence of gelatin methacryloyl (GelMA) hydrogels on atmospheric pressure plasma jet (APPJ)-treated low-density polyethylene (LDPE) surfaces after peel-off tests. **(b)** The force/width versus displacement curve obtained from the lap shear test results demonstrates strong adhesion between dried GelMA sandwiched between two APPJ-treated LDPE surfaces. No failure occurred at the GelMA/LDPE interface (n=4). **(c)** Adhesion strength of the samples, which were incubated in water for up to months and then dried and subjected to peel-off tests after 5 months. **(d)** Adhesive strength obtained from the force/displacement curve for different conditions. (UT: untreated, T: treated, S: silane functionalised LDPE, and D/R: drying and rehydration cycle. **(e)** Indentation test results compare the force required to indent as-made hydrogel coatings and rehydrated GelMA coatings after the D/R process. Data presented as mean ± SEM, n=5, *P*-values are calculated using t-tests, *****P*≤0.0001. **(f)** Water contact angle (WCA) measurements were performed on silane-functionalised LDPE surfaces. Coating 3-(Trimethoxysilyl)propyl methacrylate (TMSPMA) on APPJ-treated LDPE reduced wettability. Data presented as mean ± SEM, n=3. **(g)** The stress/displacement curves obtained from scrape tests. A set of dried samples was scratched before rehydration to investigate the effect of surface damage on the adhesion strength of the hydrogel in hydrated form.


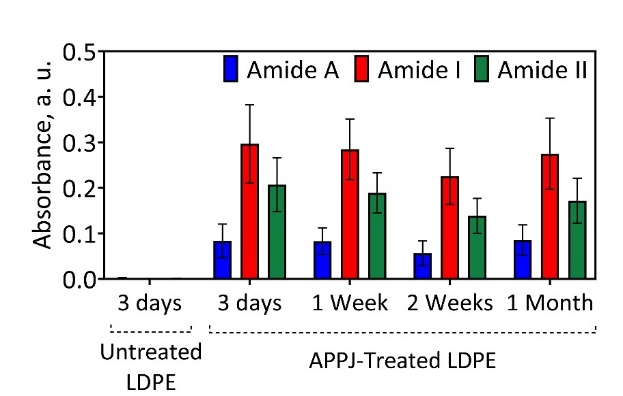


**Figure S8.** Results for static water stability tests. The intensities of amide bonds obtained from attenuated total reflectance Fourier-transform infrared (ATR-FTIR) analysis for untreated and atmospheric pressure plasma jet (APPJ)-treated low-density polyethylene (LDPE) samples coated with gelatin methacryloyl (GelMA) incubated in PBS solution for various time points. Data presented as mean ± SEM, n=3.


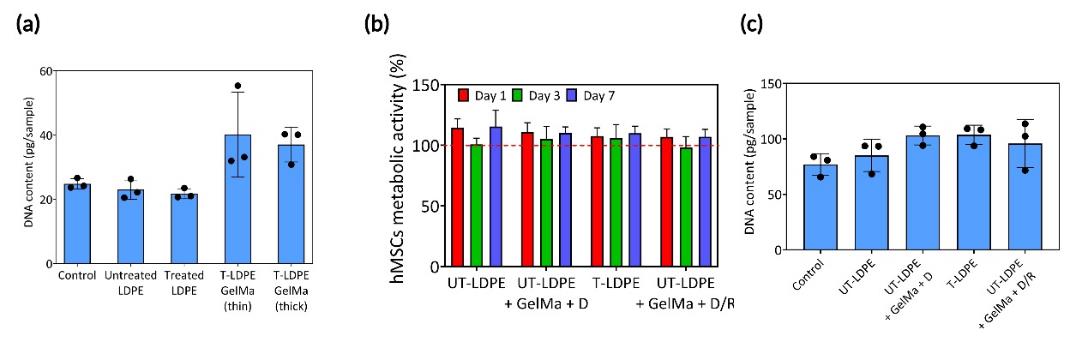


**Figure S9. (a)** DNA counting results obtained from human mesenchymal stem cells (hMSCs) after 7 days of seeding under different sample conditions (cell viability test samples), indicating the viability of the cells compared to the control. **(b)** Metabolic activity and **(c)** DNA counting results obtained from hMSCs cultured on samples after a cycle of drying and rehydration (D/R). The results demonstrate no cytotoxicity. Data presented as mean ± SEM, n=3.


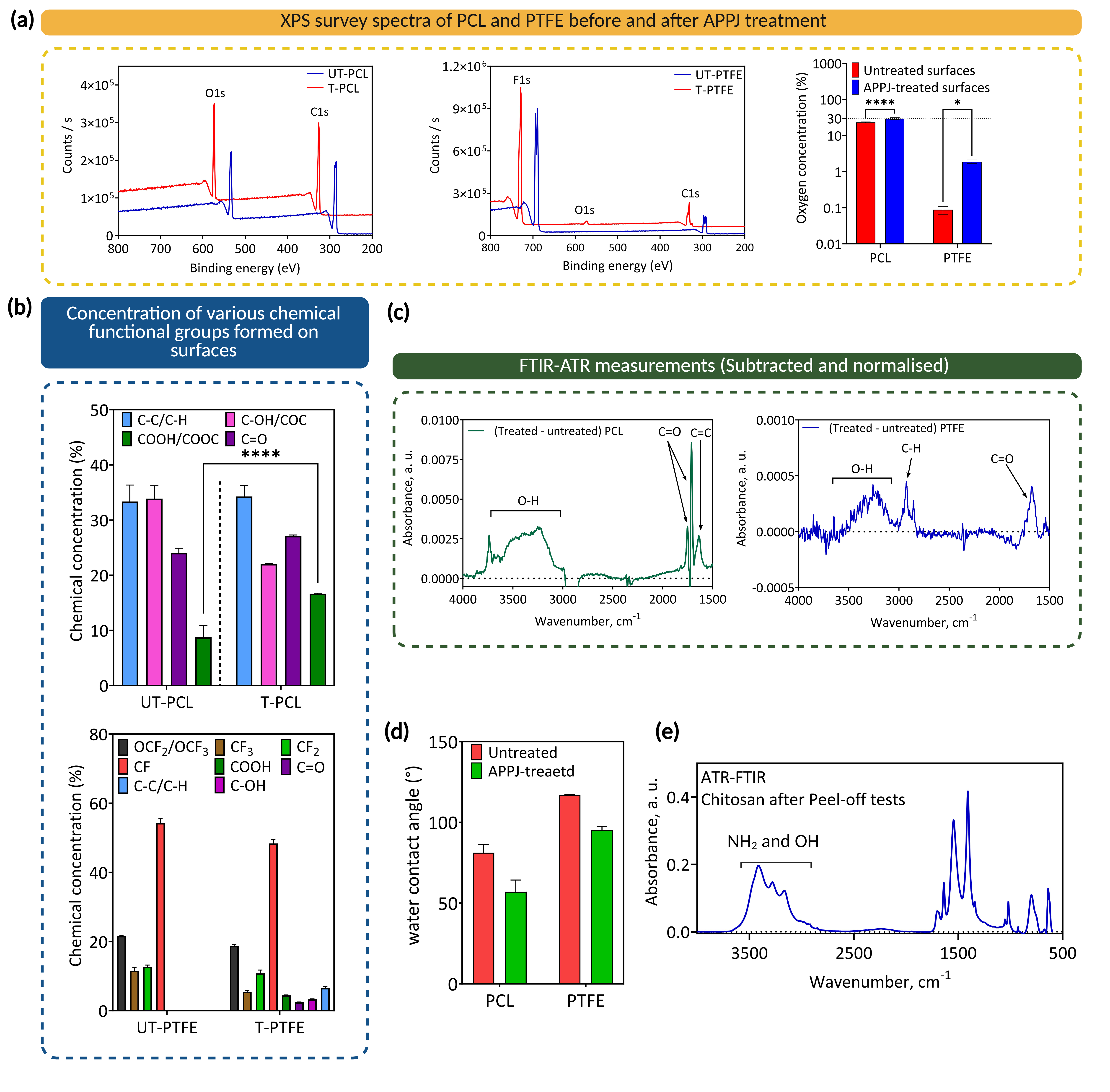


**Figure S10. (a)** X-ray photoelectron spectroscopy (XPS) survey spectra and calculated oxygen atomic concentration (atm%) of untreated and atmospheric pressure plasma jet (APPJ)-treated polycaprolactone (PCL) and polytetrafluoroethylene (PTFE) surfaces. Data presented as mean ± SEM, n=5, *P*-values are calculated using two-way ANOVA with Bonferroni correction, *****P*-value≤0.0001, **P*-value≤0.05. **(b)** The concentration of various chemical functional groups formed on PCL and PTFE after APPJ treatment. Data presented as mean ± SEM, n=3, *P*-values are calculated using two-way ANOVA with Bonferroni correction, *****P*-value≤0.0001. **(c)** The effects of APPJ treatment on the formation of oxygen-containing functional groups (OFGs) are evident in attenuated total reflectance Fourier-transform infrared (ATR-FTIR) analyses. Functional groups such as OH, C=O, and C=C were increased on PCL surfaces and COOH and C–O–C were formed, while on PTFE surfaces, functional groups like C–H, C–OH, C=O, and C–O were formed. **(d)** Water contact angle (WCA) measurements from PCL and PTFE before and after APPJ treatment. The results indicated the reduction in contact angle after plasma treatment, confirming the presence of polar functional groups after plasma treatment. **(e)** ATR-FTIR spectra obtained from chitosan-coated LDPE samples after peel-off tests. The result demonstrates the presence of chitosan on APPJ-treated LDPE after peel-off tests, highlighting the robust adhesion of chitosan.

**References**

[1] B.M. Smirnov, Theory of gas discharge plasma, Springer2015.

[2] J. Park, I. Henins, H. Herrmann, G.J.J.o.A.P. Selwyn, Gas breakdown in an atmospheric pressure radio-frequency capacitive plasma source, Journal of Applied Physics 89(1) (2001) 15-19.

[3] S.K. Alavi, O. Lotz, B. Akhavan, G. Yeo, R. Walia, D.R. McKenzie, M.M. Bilek, Atmospheric Pressure Plasma Jet Treatment of Polymers Enables Reagent-Free Covalent Attachment of Biomolecules for Bioprinting, ACS Applied Materials & Interfaces 12(34) (2020) 38730-38743.

[4] K. Yue, G. Trujillo-de Santiago, M.M. Alvarez, A. Tamayol, N. Annabi, A.J.B. Khademhosseini, Synthesis, properties, and biomedical applications of gelatin methacryloyl (GelMA) hydrogels, Biomaterials 73 (2015) 254-271.

[5] M. Zhianmanesh, A. Gilmour, M.M.M. Bilek, B. Akhavan, Plasma surface functionalization: A comprehensive review of advances in the quest for bioinstructive materials and interfaces, Applied Physics Reviews 10(2) (2023) 021301.

[6] B. Akhavan, M. Croes, S.G. Wise, C. Zhai, J. Hung, C. Stewart, M. Ionescu, H. Weinans, Y. Gan, S.A.J.A.M.T. Yavari, Radical-functionalized plasma polymers: Stable biomimetic interfaces for bone implant applications, Applied Materials Today 16 (2019) 456-473.

[7] H. Yuk, T. Zhang, S. Lin, G.A. Parada, X.J.N.m. Zhao, Tough bonding of hydrogels to diverse non-porous surfaces, Nature materials 15(2) (2016) 190-196.

[8] R. Wang, Y. Shen, C. Zhang, P. Yan, T.J.A.S.S. Shao, Comparison between helium and argon plasma jets on improving the hydrophilic property of PMMA surface, Applied Surface Science 367 (2016) 401-406.

[9] A. Ozcan, M.J.B.p. Ogun, c.s.o.o. stress, Biochemistry of reactive oxygen and nitrogen species, Basic principles and clinical significance of oxidative stress 3 (2015) 37-58.

[10] J. Gulmine, P. Janissek, H. Heise, L.J.P.t. Akcelrud, Polyethylene characterization by FTIR, Polymer testing 21(5) (2002) 557-563.
